# Whole Exome Sequencing in Healthy Individuals of Extreme Constitution Types Reveals Differential Disease Risk: A Novel Approach towards Predictive Medicine

**DOI:** 10.3390/jpm12030489

**Published:** 2022-03-18

**Authors:** Tahseen Abbas, Gaura Chaturvedi, P. Prakrithi, Ankit Kumar Pathak, Rintu Kutum, Pushkar Dakle, Ankita Narang, Vijeta Manchanda, Rutuja Patil, Dhiraj Aggarwal, Bhushan Girase, Ankita Srivastava, Manav Kapoor, Ishaan Gupta, Rajesh Pandey, Sanjay Juvekar, Debasis Dash, Mitali Mukerji, Bhavana Prasher

**Affiliations:** 1 Centre of Excellence for Applied Development of Ayurveda *Prakriti* and Genomics, CSIR Ayurgenomics Unit-TRISUTRA, CSIR-Institute of Genomics & Integrative Biology, Delhi 110020, India; tahseen.abbas@igib.in (T.A.); gaura.chaturvedi@igib.in (G.C.); rintu.kutum@igib.in (R.K.); pushkar.dakle@gmail.com (P.D.); ankita.narang86@gmail.com (A.N.); vijeta.manchanda27@gmail.com (V.M.); 2 Informatics and Big Data Unit, CSIR-Institute of Genomics & Integrative Biology, Mathura Road, Delhi 110020, India; 3 Academy of Scientific and Innovative Research, Ghaziabad 201002, India; 4 Genomics and Molecular Medicine, CSIR-Institute of Genomics & Integrative Biology, Mathura Road, Delhi 110020, India; prakrithi29@gmail.com (P.P.); pxthxk@gmail.com (A.K.P.); 5 Vadu Rural Health Program, KEM Hospital Research Centre, Pune 412216, India; ru2.patil@gmail.com (R.P.); dhiraj.agarwal99@gmail.com (D.A.); bhushangirase@gmail.com (B.G.); drankitashrivastava@gmail.com (A.S.); sanjay.juvekar@kemhrcvadu.org (S.J.); 6 Department of Neuroscience, Icahn School of Medicine at Mt. Sinai, New York, NY 10029, USA; kapoor.manav@gmail.com; 7 Department of Biochemical Engineering and Biotechnology, Indian Institute of Technology Delhi, New Delhi 110016, India; ishaan@iitd.ac.in; 8 INtegrative GENomics of HOst-PathogEn (INGEN-HOPE) Laboratory, CSIR-Institute of Genomics and Integrative Biology (CSIR-IGIB), Delhi 110007, India; rajeshp@igib.in; 9 Department of Bioscience and Bioengineering, Indian Institute of Technology Jodhpur, NH 62, Jodhpur 342037, India

**Keywords:** ayurgenomics, deep phenotypes, exomes, precision medicine, extreme phenotypes, risk stratification, exome sequencing

## Abstract

Precision medicine aims to move from traditional reactive medicine to a system where risk groups can be identified before the disease occurs. However, phenotypic heterogeneity amongst the diseased and healthy poses a major challenge for identification markers for risk stratification and early actionable interventions. In Ayurveda, individuals are phenotypically stratified into seven constitution types based on multisystem phenotypes termed “*Prakriti*”. It enables the prediction of health and disease trajectories and the selection of health interventions. We hypothesize that exome sequencing in healthy individuals of phenotypically homogeneous *Prakriti* types might enable the identification of functional variations associated with the constitution types. Exomes of 144 healthy *Prakriti* stratified individuals and controls from two genetically homogeneous cohorts (north and western India) revealed differential risk for diseases/traits like metabolic disorders, liver diseases, and body and hematological measurements amongst healthy individuals. These SNPs differ significantly from the Indo-European background control as well. Amongst these we highlight novel SNPs rs304447 (*IFIT5*) and rs941590 (*SERPINA10*) that could explain differential trajectories for immune response, bleeding or thrombosis. Our method demonstrates the requirement of a relatively smaller sample size for a well powered study. This study highlights the potential of integrating a unique phenotyping approach for the identification of predictive markers and the at-risk population amongst the healthy.

## 1. Introduction 

Precision medicine aims to stratify individuals based on endo-phenotypes and risk profiles, for early actionable interventions. Methods are still evolving to identify biomarkers corresponding to phenotypes that could enable screening of target populations, predict progression and prognosis of illness, as well as enable differential therapeutic management [1,2,3,4,5,6]. In this regard, traditional medicines have provided meaningful clinical insights for P4 medicine [7]. Most of the Genome-Wide Association Studies (GWAS) conducted for the delineation of the genetic basis of common and complex diseases are reported on single/discernible traits; in the absence of comprehensive deeper phenotypes of multisystem attributes, many of the phenotype to genotype associations still remain to be uncovered [8]. Widespread overlap of GWAS SNPs association with seemingly unrelated diseases and phenotypes [9,10] have prompted Phenome Wide Association Studies (PheWAS) in Biobanks, Electronic Health Records (EHR) as well as longitudinal cohorts [11,12,13,14,15]. PheWAS has uncovered many variants that exhibit pleiotropic effects and offers to identify disease gene networks, novel phenotypic associations of drugs side effects and leads for drug repurposing [16,17,18,19]. The success in uncovering phenotype–phenotype connectivity in PheWAS depends on the extent and diversity of captured features, as well as the co-occurrence of the phenotypes in the EHRs and cohorts [20]. Thus, even if the cohort size in PheWAS might be in millions with significant GWAS associations, the subsequent genotype–phenotype associations of the variants is observed in relatively smaller sample sizes. It is being felt that extending the GWAS to systems’ levels with deeper phenotypes and composite traits can accelerate predictive marker discoveries [8,21]. Exome sequencing of extreme phenotypes in smaller sample sizes (i.e., hundreds) is also being used as another approach to identify variants with larger phenotypic effects in single attributes or for variable clinical outcomes in diseases [22,23,24,25,26,27,28].

Ayurveda, an ancient system of Indian medicine, provides a rich repertoire of phenotypic descriptions for a comprehensive assessment of an individual’s constitution “*Prakriti*” groups [29,30]. *Prakriti* forms the basis for the prediction of the health and disease trajectory and personalized management and therapy. There are seven *Prakriti* types described on the basis of the relative proportion of three physiological entities *Vata* (V) *Pitta* (P) and *Kapha* (K) at the time of fertilization which shape the fetal development and remain invariant throughout lifetime. Amongst the seven, three are extreme *Prakriti* and are clinically distinguished on the basis of multisystem phenotypic attributes such as body frame, body build, food and bowel habits, sleep pattern, physical activity such as walking pace, strengths such as disease resistance and healing capacity, and psychological features such as memorizing power, social interaction and memory type [29,31]. The three extreme *Prakriti* groups (V, P or K) comprise nearly 10% of the population [29].

Unsupervised and supervised machine learning and advanced statistical approaches have been used to validate the existence of the *Prakriti* specific phenotype–phenotype connectivity and recapitulate the analysis through ML algorithms [32]. Studies have also provided evidence for OMIC differences at different hierarchies of genetic, epigenetic, transcriptomic, biochemical, gut microbiome, cellular level between *Prakriti* groups [32,33,34,35,36,37]. Novel markers have also been identified for hypoxia responsiveness and outcomes using this integrative approach in the north India cohort [36,38].

Differential susceptibilities of *Prakriti* groups towards diseases have been described in Ayurveda that encompass neurological disorders, the developmental anomalies for *Vata*, bleeding disorders, skin diseases for *Pitta*, and metabolic disorders such as obesity, diabetes and atherosclerotic conditions for *Kapha* [29,31].

We hypothesized that exome sequencing of *Prakriti* stratified healthy individuals defined by multi-system phenotypes could yield pleiotropic and disease associated variations differentiating amongst *Prakriti* groups as well from matched ethnic background populations. This could further enable the identification of at-risk or protective groups. Here, we report the results from an in-depth exome sequencing analysis of 144 healthy individuals of extreme constitution types across two genetically homogeneous cohorts (north India (NI), western India (KEMHRC-VADU Health and Demographic Surveillance System, Pune)) along with background controls.

Label shuffling permutation analysis revealed that the majority of *Prakriti* differentiating SNPs are true signals and random grouping could not provide these *Prakriti* differentiating variations. A core set of 115 SNPs replicate with exact patterns of allele frequency differences amongst *Prakriti* groups in both cohorts despite differences in genetic background. The majority of these “*Prakriti* replicated SNPs” are expression QTLs, implying their functional significance. In each cohort, some of these *Prakriti* replicated SNPs also significantly differed from respective background populations (which would comprise a mix of all *Prakriti* types). The integration of this method could thus assist in the identification of susceptible groups from healthy populations. From this set, we found novel leads in *IFIT5* and *SERPINA10* genes with *Prakriti* that could explain differential disease trajectories.

*Prakriti* differentiating SNPs from both the cohorts map to genes of distinct biological processes, such as anatomical structure and organ development, cell morphogenesis, cell adhesion, extracellular matrix organization, transport and signal transduction, biosynthetic metabolic processes, immune response and inflammation, sensory perception, behavior, and fear response. Some of the biological processes are enriched in specific *Prakriti* comparisons of one cohort, such as hemostasis and wound healing in the north India cohort, and the sensory perception of taste, pain and sound in the Vadu cohort. We also observed *Prakriti* differentiating variations mapping to anatomical, metabolic, psychological traits in the GWAS catalog. In addition to this, SNPs are associated with measurements such as lipid or lipoprotein, hematological and inflammatory markers. Even though, these measurements do not form the basis of *Prakriti* stratification. We also observed a substantial overlap with pleiotropic leads reported in the UK Biobank. This study provides a wealth of informative SNPs that could be useful in estimating predictive risk scores and a phenotypic method for risk stratification amongst healthy individuals for early interventions that would not have been possible without phenotypic stratification.

## 2. Materials and Methods 

### 2.1. Sample Description

Exome study was carried out on healthy subjects of extreme *Prakriti* groups identified from our two earlier studied genetically homogeneous cohorts (NI [32,33,36,38] and Vadu [32]). Extensive protocols have been followed for recruitment of subjects, clinical phenotyping, classification into predominant groups as well as establishment of genetic homogeneity, as have been described in earlier papers [32,33]. Briefly, the extreme *Prakriti* *groups* comprise 10% of the studied population, belong to the age group of 18–40 years, exhibit differences with respect to ~150 multisystem features that include anatomical and physical and physiological attributes, as well as psychological and other responses [39]. Genetic homogeneity of the study cohorts and its relatedness to diverse Indian populations was affirmed by principal component analysis using a set of 17,675 SNPs that overlapped with the Indian Genome Variation Consortium (IGVC) diversity panel [40]. The study has been carried out as per protocols approved by institutional human ethics committee at CSIR-Institute of Genomics and Integrative Biology, Delhi and KEM Hospital Research Center, Pune, India.

The sample set includes 108 healthy individuals (18 each of *Vata* (V), *Pitta* (P), *Kapha* (K) in NI & Vadu cohort each) Additionally Indo-European (IE) control subjects (C) of heterogeneous phenotypes not classified by Prakriti, 18 each for NI and Vadu cohorts, were sequenced to get background allele frequencies.

### 2.2. Whole Exome Sequencing (WES) and Variant Calling

Exome sequencing of 144 healthy subjects was carried out on Illumina HiSeq2000 platform using standard methods. The sample reads were aligned to the genome (reference GRCh37) using BWA version 0.7.4 (http://bio-bwa.sourceforge.net, accessed on 10 October 2020), converted to BAM format and indexed using SAMtools (version 0.1.18, https://samtools.github.io, accessed on 10 October 2020). Post alignment, the samples were marked for duplicates and realigned. The variants were called using Haplotype Caller (GATK version 4.1.2). Vcftools 0.1.12 was used to convert genotypes in VCF format to Plink format for statistical analysis. Variants were annotated using Annovar [41] with novel variants indicated by their chromosomal position in version GRCh37. Additionally, variants with less than 50% genotyping call rates were removed from each pairwise comparison prior to analysis. Three pairwise comparisons; *Vata* vs. *Pitta* (Vvs.P), *Pitta* vs. *Kapha* (Pvs.K) and *Vata* vs. *Kapha* (V vs. K) were carried out to identify differentiating variants using Fisher’s exact test (*p*-value < 0.05) implemented in PLINK (v1.7). To assess whether the differences were *Prakriti* specific, permutation analysis was carried out by randomly shuffling the *Prakriti* labels and comparing Fisher’s *p* value from the permuted set with that of the original set for each of the SNPs. A range of permutations from 10,000, 80,000 and 1 lakh times for each SNP in NI and Vadu cohort, respectively, was carried out using a custom script in R (v4.0.2) and bash. Post permutation, the SNPs that were present in lower 5% distribution of *p*-values of the permuted set were retained. Profiles for significant SNPs were indicated on the basis of alternate allele frequencies calculated using a perl script, for example, in a V vs. K comparison, a profile of V+K- represents higher alternate allele frequency in *Vata* compared to *Kapha* in both cohorts, despite differences in frequency in the background population between the cohorts.

### 2.3. Mapping and Enrichment Analysis for Disease/Traits Associations 

We queried the *Prakriti* differentiating SNPs for disease associations using GWAS catalog v1.0.2 (https://www.ebi.ac.uk/gwas/, downloaded on 30 September 2020). The catalog houses 132050 SNPs associated with 4634 Disease/Traits. CrossMap [42] was used to convert the genome build of exome coordinates (from GRCh37 to GRCh38) to match GWAS data. SNP profiles were represented with respect to the risk allele in the GWAS catalog. The disease/traits from GWAS catalog were mapped to their parent term from EFO (Experimental Factor Ontology (https://www.ebi.ac.uk/ols/ontologies/efo), accessed on 3 November 2020). EFO has 6340 Disease/Traits from GWAS catalog mapped to 17 parent terms. In order to verify whether the presence of GWAS associated SNPs in our data was by chance, we randomly picked 6000 SNPs (equivalent to significant SNPs from Vadu Cohort) from the pool of ~2 lakh exonic SNPs from Vadu Cohort and intersected it 1000 times with GWAS catalog. The median value from 1000 random intersections was tested against the original GWAS SNP numbers from *Prakriti* differentiating list using Chi-square test. We computed whether the disease/traits associated with each *Prakriti* group showed differential enrichment. We used the entire dataset of GWAS catalog as background to assess enrichments. This was carried out using Fisher’s exact test function in R v4.1.1.

### 2.4. Multi-System Phenotype Association Analysis Using GeneAtlas

*Prakriti* differentiating SNPs were queried for multi-system phenotypes association in GeneATLAS database [43] that houses associations between hundreds of traits and millions of variants using the UK Biobank cohort. We downloaded the summary statistics for different traits using a bash script provided by the GeneATLAS (http://geneatlas.roslin.ed.ac.uk/downloads/?traits=0, accessed on 10 October 2020). The effect sizes and *p* values were extracted for different health and metabolic traits related GWAS analysis.

### 2.5. Replication Analysis of Prakriti Differentiating SNPs

The extent of replication was assessed at three different levels: (1) overlapping genes with identical and/or different SNPs as well as profiles; (2) identical SNPs having similar and/or different profiles; (3) identical SNPs with exactly matching profiles in both cohorts which we will be referring to as “*Prakriti* replicated Profile SNPs”. For the third group of identical SNPs, pairwise comparisons of each of the *Prakriti* groups with the background controls (V vs. C, P vs. C, K vs. C) were also carried out using Fisher’s exact test in both cohorts. 

### 2.6. Power Analysis

Since the study involves extreme and composite phenotypes that comprise 10% of the population, we anticipate adequate power in smaller sample sizes. As of now, no estimates of sample sizes for an adequately powered study on healthy individuals, and that too of extreme and composite types, are available. Therefore, we estimated the power of the study based on the allele frequencies from our data on two cohorts from each comparison group (e.g., V vs. K). We quantified power using a “power.fisher.test” function of statmod R-package (v4.0.2) [44]. Power estimations were done using simulations performed with increasing sample sizes. Initially original sample numbers used for frequency estimation were used, followed by a stepwise increase in sample numbers (18, 50, 100, 500, 1000, 10,000) with alpha of 0.05.

## 3. Results

### 3.1. Genetic Differences amongst Healthy Prakriti Types Remain Significant after Permutation Analysis

Exome sequencing datasets provided a total of 229,029 and 254,200 variants in NI and VADU cohorts, respectively. Fisher’s exact test to identify SNPs provided a set of significant SNPs. Permutation analysis of shuffled samples from *Prakriti* groups provides more significant *p* values at 80,000 permutations compared to 10,000 iterations (Figure S1A,B). Based on this we fixed our iterations to 80,000 to select a cutoff of significant SNPs from each comparison. We identified 5925 unique variations (3482 genes) in NI and 6103 (3554 genes) in VADU to differentiate between *Prakriti* types (Table S1). The differentiating variants show near similar distributions across the genic region in both cohorts ruling out any bias in sequencing. As anticipated, about 50% of the differentiating variations map to exonic regions with a significant fraction in 3’UTRs (Figure S2A,B).

### 3.2. Distinct Enrichment of Biological Processes in Prakriti Groups: Similar Patterns across Both Cohorts

We next analyzed the biological basis of phenotypic variability amongst *Prakriti* types. Gene Ontology (GO) analysis of genes with differentiating SNPs in the three *Prakriti* groups comparisons reveal significant enrichments (*p*-value < 10^−2^ without correction) of biological processes in both cohorts. We found certain enriched biological processes to be differentiating between all *Prakriti* types, such as processes related to actin cytoskeleton organization, cell adhesion, cell–cell signaling, cell morphogenesis, extracellular matrix organization, nervous system development, nephron development.

Certain biological processes were found to be enriched in one *Prakriti* only. For instance, a Type 1 interferon signaling pathway, Natural killer cell activation involved in immune response and Tricarboxylic acid cycle were enriched in VP and PK (NI cohort), and the Negative regulation of leukocyte mediated immunity and Response to Interferon gamma were enriched in VP and PK (Vadu cohort). Metabolism and biosynthetic related processes such as the Aldehyde biosynthetic process and neuron migration were enriched in the VK and PK (Vadu cohort) comparison groups and the integrin mediated signaling pathway was enriched in VK and PK (NI cohort).

Additionally, few enriched biological processes were found exclusively in only one *Prakriti* comparison in either or both of the cohorts. For instance, (a) PvsK comparison: adaptive thermogenesis, diet induced thermogenesis, and Type IV hypersensitivity in the Vadu cohort; blood coagulation, wound healing, hemostasis, and regulation of body fluid levels in the NI cohort; somatic muscle development in both the cohorts; (b) VvsK comparison: visual behavior, fear response, cellular glucose homeostasis, Thyroid-stimulating hormone-secreting cell differentiation, cellular response to follicle-stimulating hormone stimulus and Leutinizing hormone stimulus, sensory perception of bitter taste, cardiac muscle development and oscification in the Vadu cohort; keratinization and aldosterone secretion in the NI cohort; and brain development in both the cohorts; and (c) VvsP comparison: walking behavior, determination of bilateral symmetry, and response to histamine in the Vadu cohort; and behavioral fear response, histamine secretion, lymph circulation, Hormone secretion and transport, and the regulation of bone remodeling in the NI cohort (Table S2). 

### 3.3. Significant Enrichment of Prakriti Differentiating SNPs for Variants with Common and Complex Diseases 

**Figure 1:**
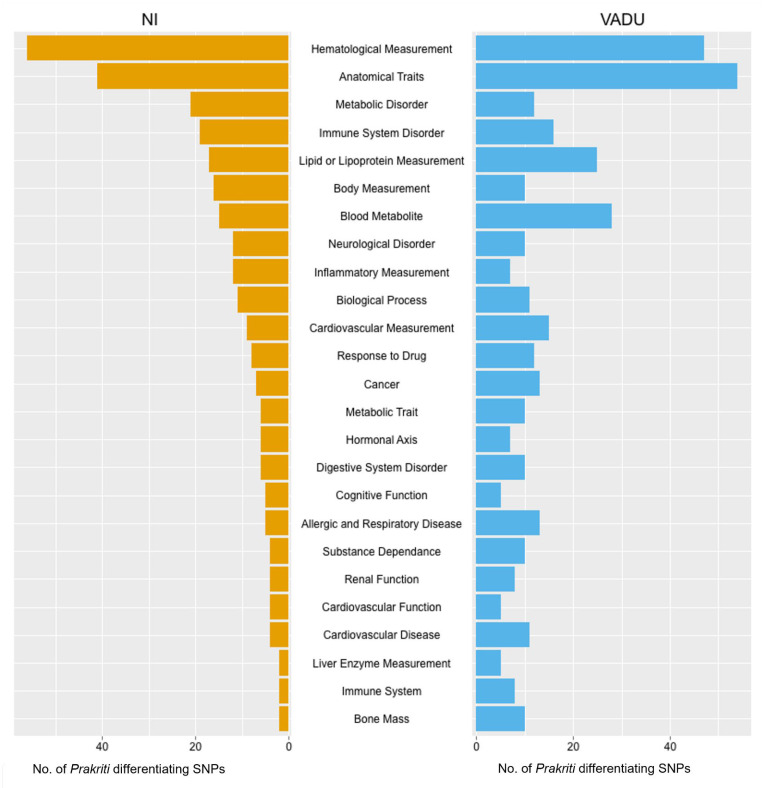
Barplot showing number of *Prakriti* differentiating SNPs associated with top 25 GWAS disease/traits Parent terms in NI and Vadu cohort.

Extreme *Prakriti* groups differ with respect to multisystem traits and are differently predisposed to diseases. If SNPs that differentiate cases from controls also differentiate healthy subjects stratified phenotypically, then conditioning association studies with these phenotypes could help in identifying novel leads. We, therefore, explored if the *Prakriti* differentiating SNPs were associated with disease/traits in the GWAS catalog. We found 289 and 313 SNPs from the NI and VADU cohorts, respectively, to be associated with 287 & 309 GWAS diseases/traits (Table S3). Interestingly, these SNP numbers are highly significant when compared to any random SNP set intersection with the GWAS catalog (*p*-value < 2.537 × 10^−15^). Broadly, the *Prakriti* differentiating variants are also associated with a) anthropometric traits, such as BMI, waist-to-hip Ratio (WHR), and arm circumference, b) disease categories such as metabolic disorders, neurological disorders, allergic and respiratory diseases, and c) parameters such as hematological measurements, blood metabolites and inflammatory measurements (Figure 1 and Figure S3A,B). The majority of these *Prakriti* differentiating GWAS SNPs are also cis-eQTLs in GTEx data which allowed us to anchor the associated variants with the expression across tissues and different *Prakriti* types (Table S4). 

### 3.4. Enriched Disease/Traits Associated with Prakriti Differentiating Genetic Variations Enable Risk Stratification

**Figure 2:**
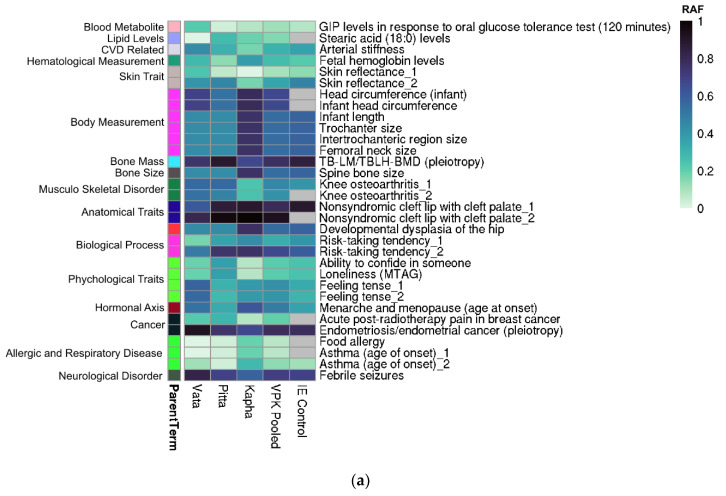

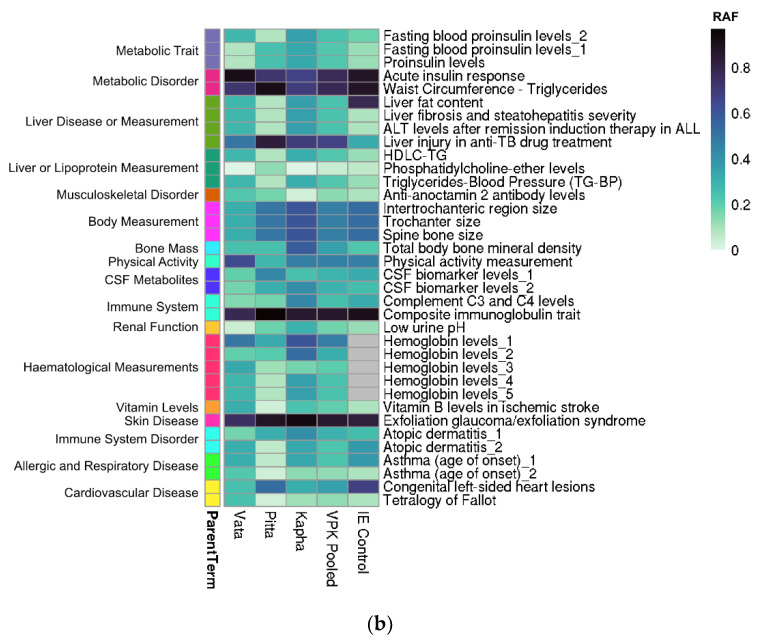
Heat Map showing pattern of risk allele frequencies (RAF) for enriched GWAS diseases/traits across *Prakriti* groups in (**a**) NI and (**b**) Vadu cohort. Enrichment was performed for V, P, K differentiating diseases/traits against GWAS catalog. In case a disease/trait has more than 1 SNP associated, it is indicated by underscore followed by number. RAF increases as we go from lighter towards darker color. Cells with grey color denote RAF is unavailable for that disease/trait. Disease/Traits on right side have been grouped into Parent Term (left side grouping), as mentioned in GWAS catalog.

We next evaluated the enrichment of diseases/traits associated SNPs in Prakriti groups using the Chi-square test. (Table S5). Certain traits were found to be enriched in similar *Prakriti* in both cohorts, even though the genes associated with them were different. Hemoglobin level SNPs were found to be enriched in the “P” group, whereas body measurement traits such as spine bone size, trochanter, Intertrochanter size were enriched in the “K” group in both cohorts (Figure 2).

We wanted to identify if the subgroups from our healthy cohort are predisposed for risk or protection against diseases/traits reported in the GWAS catalog. For this, reference/alternate alleles of *Prakriti* differentiating variations in our data were matched to the corresponding risk alleles from GWAS catalog (Figure S4A,B). We found that *Prakriti* groups show differential risk for certain broad Parent Term categories in both cohorts.

For instance, in the NI cohort, (a) the “V” *Prakriti* group were enriched in SNPs associated with high risk allele frequency for GIP levels in response to an oral glucose tolerance test, febrile seizures and psychological traits such as feeling tense; (b) the “K” group with high risk to body measurement traits such as WHR, BMI, asthma (age of onset) and low risk to skin reflectance, post-radiotherapy pain (breast cancer) and psychological traits such as ability to confide in someone and feeling tense; (c) the “P” group for high risk allele frequency of SNP associated with stearic acid levels and low risk for body measurement traits such as WHR (Figure 2a, Table S5). 

In the Vadu cohort we observed enrichment in (a) the “V” *Prakriti* group for traits such as acute insulin response, CSF biomarker levels, body measurement trait such as spine/trochanter/intertrochanteric size/infant head circumference, associated with lower bone size; (b) lower risk associated SNPs were in the “P” group for asthma (age of onset), liver disease such as liver fibrosis, liver fat content and drug response measurements such as ALT levels after ALL; (c) the “K” group was associated with a higher risk for anatomical traits such as spine bone size, trochanter/intertrochanteric size and diseases such as liver disease and skin disease in comparison to the other two groups (Figure 2b, Table S5).

### 3.5. Risk Stratification amongst Healthy Individuals: Potential for Early Identification 

Extreme *Prakriti* groups differ with respect to the enrichment of variants associated with diseases/traits. However, it would be interesting to identify whether these variants also distinguish the *Prakriti* groups from the background population, that is a pool of all the *Prakriti* groups. For this, we compared the GWAS risk allele frequency (RAF) within the *Prakriti* groups as well as with the background (Indo-European, IE) control, and prioritized SNPs for which GWAS RAF not only differs between *Prakriti* groups, but also differentiates significantly (*p* < 0.05) from the background control for each cohort (NI and Vadu), separately (Table 1 and Figure S5A,B). This could thus help in identifying susceptible/protected subgroups within a population.

### 3.6. Multi-System Phenotypic Associations of Prakriti Differentiating Variants in GeneATLAS

**Figure 3:**
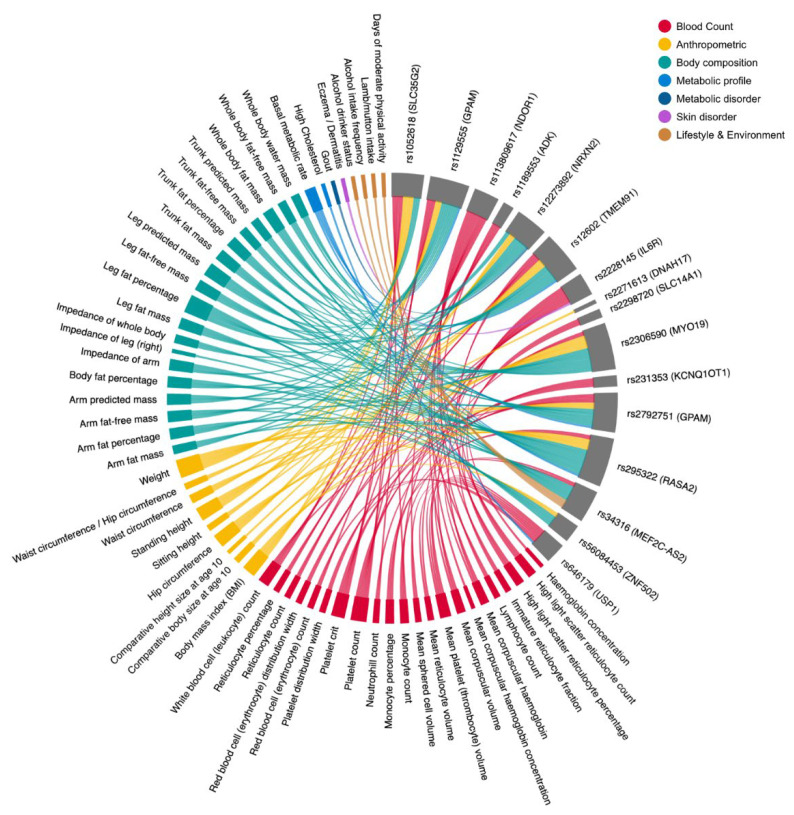
Chord Diagram showing 16 common GWAS SNPs across both cohorts (NI and Vadu) associated with multiple phenotypes (*p* < 10^−6^) in the UKBB cohort retrieved from GeneATLAS. The ribbons connect the phenotype to the differentiating common GWAS SNPs. Phenotypes broadly fall in seven groups: Blood count, Anthropometry, Body composition, Metabolic profile, Metabolic disorder, Skin disorder, Lifestyle and Environment. Right side (gray color bars) denotes the GWAS SNPs that are shared between the cohorts. Width of the gray bars depends upon the number of associated phenotypes. Colors on the left side depict a broader phenotype category.

To study the potential pleiotropic consequences of *Prakriti* differentiating variants associated with diseases/traits, we queried the GeneATLAS which contains variants associated with UK Biobank (UKBB) traits. We found 277 and 306 Prakriti differentiating SNPs from the NI and Vadu cohorts, respectively, to be associated (*p* < 0.05) with 776 UKBB phenotypes in the GeneATLAS (Table S6, Figure S6). Interestingly, we found similar associations with anthropometric traits, hematological and body composition, and other metabolic traits such as BMI, basal metabolic rate (BMR), diabetes, and hypertension, as found from GWAS catalog. We found highly significant (*p* < 10^−6^) phenotypic associations for five *Prakriti* replicated profile SNPs. These were rs1189553 (*ADK*, P vs. K), rs295322 (*RASA2*, P vs. K), rs56084453 (*ZNF502*, P vs. K and V vs. P), rs12602 (*TMEM91*, V vs. K) and rs2298720 (*SLC14A1*, V vs. P), along with other SNPs from genes such as *GPAM* and *IL6R*, and are shown in the chord diagram (Figure 3).

### 3.7. Similar Patterns of Exonic Differences: Identification of Prakriti Replicated Profile SNPs across Both Cohorts

A total of 993 genes with 480 identical SNPs were observed to be replicated between *Prakriti* groups across both cohorts (Table S7). Noteworthy, 115 identical SNPs from 106 genes have similar pattern of frequency differences between the *Prakriti* groups, e.g., in a V vs. K comparison, a profile of V+K- represents a higher alternate allele frequency in V compared to the K group in both cohorts (Table S8). Amongst these replicated profile SNPs, eight replicated SNPs in *ANKLE1, ZNF502, RASA2, CTU1, ADK, SLC14A1, TMEM91* and *HLA-DQB2* are associated with disease/traits in the GWAS catalog (Table S3). 

### 3.8. Prakriti Replicated Profile SNPs Significantly Differ from Background Population

For 115 identical SNPs, the pattern of the frequency difference between *Prakriti* groups is exactly the same in both cohorts (Table S8). However, in the background population, their allele frequency differs between the cohorts. This might be due to heterogeneity in proportions of different *Prakriti* types in background controls. Comparisons of *Prakriti* groups with background controls (V vs. C, P vs. C, K vs. C) could reveal disease susceptible groups amongst healthy populations. We found 36 SNPs in Vadu and 28 SNPs in NI, with *Prakriti* groups significantly differentiating (*p* < 0.05) from the background control (Table S8). Amongst these are 5 disease/trait associated SNPs, namely rs1189553 (ADK (lymphocyte counts); K+C-, P+K-), rs2298720 (SLC14A1 (mean corpuscular hemoglobin concentration); P-C+, K-C+, V+P-), rs12602 (TMEM91; V-C+, V-K+), rs295322 (RASA2 (lymphocyte counts associated); K+C-, P-K+) and rs56084453 (*ZNF502* (BMI associated); P-C+, P-K+). *Prakriti* based phenotyping may help in screening these individuals. 

### 3.9. Novel Leads from Prakriti Replicated Profile SNPs Confer Differential Disease Trajectories

We further assessed the potential functional consequences of our replicated profile SNPs set in order to identify novel leads that could be used for further validation. Out of 115 replicated profile SNPs, 92 SNPs regulated the expression of either a nearby gene or its own expression, thus acting as an eQTL in several tissues, as shown in the GTEx v8. Amongst these 92 eQTLs, only 6 SNPs (rs1189553 (*ADK*), rs2298720 (*SLC14A1*), rs8100241 (*ANKLE1*), rs12602 (*TMEM91*), rs12983578 (*CTU1*) and rs56084453 (*ZNF502*)) are associated with a disease or trait in the GWAS catalog (Table S9). The remaining SNPs could be potential candidates for biologically meaningful genetic variations underlying inter-individual variability amongst *Prakriti* types.

**Figure 4:**
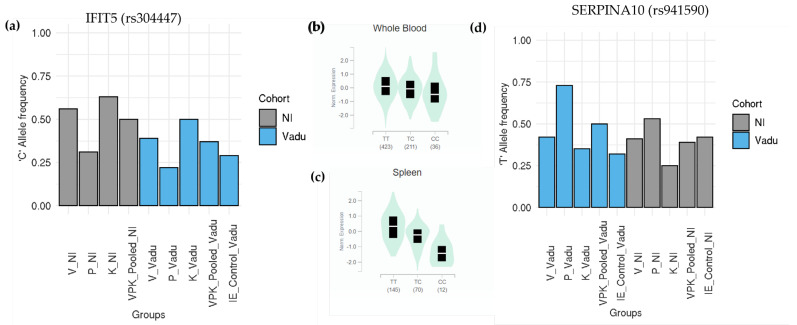
(**a**) Bar plot for alternate allele frequency in missense SNP rs304447 in *IFIT5* gene across *Prakriti* groups, IE Control and Pooled population (V, P, K combined) from NI and Vadu cohort. Frequency of the alternate allele “C” is significantly lower in the *Pitta* group than *Kapha* (*p* < 0.01 NI, *p* < 0.02 Vadu). Background control frequency of rs304447 could not be obtained for NI cohort. (**b**,**c**) Violin plots for normalized expression across Whole Blood & Spleen from GTEx v8. Alternate allele “C” is linked with lower *IFIT5* expression. (**d**) Bar plot for reference allele frequency “T” in missense SNP rs941590 in *SERPINA10* gene across *Prakriti* groups, IE control and Pooled population (V, P, K combined) from NI and Vadu cohort. Frequency of the reference allele “T” is significantly higher in the *Pitta* group than the *Kapha* (*p* < 0.03 NI, *p* < 0.002 Vadu). This allele also significantly differentiates *Pitta* group from background control.

We demonstrated examples of two replicated SNPs from *IFIT5* and *SERPINA10* genes. *IFIT5*, a member of the IFN-induced protein with tetratricopeptide repeats which enhances the innate immune response during an RNA virus infection [45], and differs significantly between *Pitta* and *Kapha*. The missense variant rs304447 is an eQTL in the GTEx with prominent effect sizes in diverse tissues like whole blood and the spleen (Figure 4b,c). The alternate allele “C” of rs304447 that associates with lower expression is significantly lower in *Pitta* compared to *Kapha* (Figure 4a). We also found a missense SNP in the *SERPINA10* gene that was identified as a protein QTL and has not been associated with a disease/trait in the GWAS catalog (Figure 4b,c). *SERPINA10* is a protein Z-dependent protease inhibitor which inhibits activity of the coagulation protease factor Xa in the presence of PROZ, calcium and phospholipids [46]. It also inhibits factor XIa in the absence of cofactors. In a study done by Yao et al. for the identification of plasma protein QTL in cardiovascular disease, the missense variant rs941590 explained 32% of the inter-individual variation in *SERPINA10* levels [47]. In this study, individuals with homozygous “TT” genotypes have been shown to have higher *SERPINA10* levels in blood plasma. In our data, this SNP significantly differs between *Pitta* and *Kapha* in both cohorts (*p* < 0.03 NI, *p* < 0.002 Vadu) with *Pitta* individuals having higher frequency of the reference allele “T” than *Kapha* individuals (Figure 4d). Interestingly, this allele not only differentiates between *Prakriti* groups, but also differentiates them from the background control, with *Pitta* individuals having a significantly higher “T” allele frequency (*p* < 0.001) in the Vadu cohort (Figure 4d). 

## 4. Discussion 

Extreme phenotyping has evolved as a strategy to homogenize subgroups based on phenotypes, thus requiring smaller sample sizes to attain sufficient power. This approach has helped in identifying protective or deleterious variations which usually remain unidentified in complex diseases due to phenotypic heterogeneity in case/controls. In the present study on exome sequencing of extreme constitution types, we have used an approach based on *Prakriti* classification described in Ayurveda, for resolving phenotypic heterogeneity amongst healthy individuals. 

We hypothesized that the *Prakriti* classification of normal healthy individuals using this method could lead to the identification of genes and genetic variations corresponding to functionally important variations and disease predictive markers.

In this study, we performed 80,000 label shuffling permutations in *Prakriti* samples in each cohort to remove any false positive associations with *Prakriti*. By doing this, we observed 115 differentiating SNPs to be replicating with the exact pattern of allele difference in *Prakriti* comparisons in both the cohorts. Interestingly, our *Prakriti* stratified data were significantly enriched for disease/traits associated variations as compared to any random set of equivalent numbers of SNPs. In addition to clinical parameters used for the *Prakriti* assessment that involve body frame and other anatomical traits, Prakriti differentiating variations associated with parameters such as hematological, inflammatory traits, blood metabolites, and diseases, such as neuro-psychological, metabolic and immune related, were also found in our data. We also observe genes to be enriched in biological processes in Prakriti comparisons, such as cell adhesion, extracellular matrix organization, cellular transport and signaling, viral immune response, and the regulation of body fluid levels. Though these could explain the modern biological basis of Vata, Pitta, Kapha functions, phenotypes and disease susceptibilities described in Ayurveda, these parameters do not form the basis for Prakriti stratification. Thus it would be interesting to perform Polygenic Risk score (PRS) analysis for these traits or processes in order to distinguish these extreme phenotype groups more. 

Some of the disease/traits associated risk alleles were enriched in specific Prakriti groups, such as body measurement traits (trochanter size, spine bone size, head circumference) in Kapha and hemoglobin levels in Pitta in both the NI and Vadu cohort, psychological traits such as feeling tense in Vata in the NI cohort, liver fat content and cirrhosis in Pitta, and metabolic traits such as proinsulin levels in Vata in the Vadu cohort. These enriched diseases/traits resonate with phenotypic and disease susceptibility descriptions for Prakriti groups. Our previous study on biochemical parameters in healthy individuals classified by Prakriti in the NI cohort has also shown hemoglobin levels to be high in Pitta males as compared to other groups.

Although these diseases/traits associated SNPs differentiate between Prakriti groups, we wanted to identify whether they also differentiate Prakriti groups from the population background which could lead to the identification of susceptible/ protected sub- phenotype populations amongst the healthy (Table 1). Noteworthy amongst the examples are rs738409 (PNPLA3, associated with cirrhosis, risk in Pitta), rs11603334 (ARAP1, fasting blood proinsulin levels, risk in Kapha), rs1552224 (ARAP1, acute insulin response, risk in Vata) and rs682331 (NIBAN1, obesity related traits) in the Vadu cohort, and rs699 (AGT, mean arterial pressure, risk in Vata) and rs2792751 (GPAM, HDL cholesterol levels, Apolipoprotein A1 levels, risk in Pitta) in the NI cohort (Table 1). 

PNPLA3 or adiponutrin is an enzyme known to have hydrolase activity towards triglycerides and retinyl esters. Knockdown experiments in mice have shown that the accumulation of PNPLA3 per se causes fatty liver, and depletion of the protein is a potential strategy for therapeutic intervention [48]. A missense variant rs738409 (I48M, C > G) in this gene has been reported to be associated with risk for Non-Alcoholic Fatty Liver Disease (NAFLD), total triglyceride levels, liver fat content, liver fibrosis and steatohepatitis severity, T2D and ALT levels. This variant is a known drug target and significantly features in studies in four large PheWAS cohorts with extensive health records (700,000) from 23 and Me, UK Biobank, FINRISK, CHOP [18]. In these studies, the “G” allele of *PNPLA3* has been implicated as a potential drug target for alcohol-related cirrhosis, Non-Alcoholic Fatty Liver Disease (NAFLD) and hepatic steatosis. We observed the “G” allele (rs738409), that is responsible for the accumulation of *PNPLA3* protein, to be significantly lower in *Pitta* as compared to *Kapha* individuals in the Vadu cohort (Figure S5B). This allele also differentiates between background controls and *Pitta* individuals. Additionally, in PheWAS studies, pleiotropic associations of the “G” allele with a decreased risk for acne, gout and gallstones have also been shown. Differential disease susceptibilities with respect to metabolic conditions are corroborating with Ayurvedic descriptions.

Since *Prakriti* encompasses multi-system phenotypes, we wanted to study if differentiating SNPs could have pleiotropic effects. A large number of GWAS SNPs in our dataset are reported to have pleiotropic effects in the UK Biobank cohort. For example, the *Pitta Kapha* replicated profile GWAS SNPs in *RASA2, ADK* and *CTU1* have an association with immuno-metabolic traits and diseases, blood and coagulation related traits/diseases such as venous thromboembolic disease (Table S6). In addition to clinically observable phenotypes, such as anatomical, body measurements associated with disease/trait/phenotype, variations underlying traits, such as hematological, biochemical and metabolic, were also reported. The pleiotropic effects of differentiating variants could explain the multisystem differences amongst *Prakriti* groups. 

We further looked for *Prakriti* differentiating SNPs with replicated profiles in both cohorts. Amongst these, we found two SNPs in the *ZNF502* and *IFIT5* genes and one missense SNP in the *SERPINA10* gene that are discussed further. The *ZNF502* missense SNP rs56084453 is an eQTL with the reference allele “A”, significantly associated with its lower expression in GTEx tissues such as the spleen and whole blood (Figure S7). The functional knockdown of ZNF502 has been reported to limit the replication of the human respiratory syncytial virus (RSV) [49]. Thus, “A” allele of rs56084453 which is fixed in *Pitta* in both the cohorts (Figure S7) might confer protection in *Pitta* group from recurrent viral infection as compared to other two *Prakriti* groups. It would be noteworthy to mention that another replicated SNP rs304447 in the *IFIT5* gene exhibits a similar pattern of association amongst *Prakriti* groups. *IFIT5* is a member of the IFN-induced protein with tetratricopeptide repeats, and enhances the innate immune response during an RNA virus infection. The alternate allele “C”, associated with lower expression, is significantly lower in *Pitta* as compared to the *Kapha* group. (Figure 4a–c). Both these observations suggest that variations associated with *Pitta Prakriti* could be involved in an enhanced antiviral response and *Pitta Prakriti* individuals might counter viral infections more readily. Another important lead from our *Prakriti* replicated profile set is a missense variant rs941590 in the *SERPINA10* gene. Protease inhibitors from the serpin superfamily regulate coagulation and fibrinolysis [46]. *SERPINA10* has an important role in hemostasis and is involved in processes such as wound healing, coagulation. This SNP differentiates *Pitta* from *Kapha* with *Pitta* individuals having a significantly higher “T” allele frequency. This SNP has been reported as a plasma protein QTL (pQTL) in a GWAS of Framingham Heart Study that involved 6861 participants and explained 32% of inter-individual variations in *SERPINA10* levels [47]. It has not been reported in the GWAS catalog, although the haplotype containing this variant has been previously reported to be associated with a family history of venous thrombosis [50]. We have earlier reported differences in the von Willebrand factor (*VWF*) that also mapped to this pathway between *Pitta* from *Kapha* [38] and could explain thrombotic outcomes in high altitude hypoxic conditions in *Kapha* individuals [36,38]. Similar to *VWF, SERPINA10* missense SNP could be important to test in diseases where there are patient stratification needs for predicting outcomes such as bleeding or thrombosis. In general, functional enrichment analysis of *Prakriti* differentiating genes has shown an enrichment of biological processes, such as the regulation of viral release from the host cell, negative regulation of viral life cycle, regulation of body fluid levels, wound healing, hemostasis and coagulation (Table S2) in the Vadu and NI cohorts. The genes discussed above, such as *ZNF502*, *IFIT5* and *SERPINA10*, are a part of these enriched processes and have an effect on gene expression or protein levels. It would be interesting to look for variations in other *Prakriti* differentiating genes from these enriched processes for their collective downstream effects through computing the Polygenic Risk score (PRS).

A limitation of the study may be the low sample size. Since this is a pilot study, we calculated its power based on allele frequencies and increasing sample sizes, and estimated the sample requirements for the better designing of adequately powered studies in the future (Table S10, Figure S8A,B). Using different sample sizes, simulation studies based on the frequency differences of the differentiating SNPs, we estimate substantial power if the studies are conducted even in 50 samples of each group (alpha = 0.05) in both the cohorts. Given the composite nature and phenotypic architecture of *Prakriti*, we might need to evolve new methods for the estimation of power, and our observations, though preliminary, would be useful for such calculations.

Our study has revealed that a wealth of known disease/trait associated variants segregating healthy individuals in a phenotype based manner could lead to risk group identification. It also highlights the need for using *Prakriti* phenotype scaffolds to identify novel genetic associations in future case-control studies for complex diseases. Eventually, these leads could also serve as important biomarkers for pre-screening individuals prior to exposure, and for targeted interventions. Conditioning genetic association with *Prakriti* information in rheumatoid arthritis and pharmacogenetics have earlier highlighted the merit of such an approach [51].

## 5. Conclusions

Exome sequencing of healthy individuals of extreme *constitution* types has provided (a) potential leads to predict differences in disease progression, response to environmental triggers and therapeutic interventions and (b) a clinical method for phenotypic stratification of a population into homogeneous groups. The merit of this approach includes clinical phenotypes as a readout for biological variability underlying *Prakriti*, which could enable the discovery of informative variants in comparatively smaller sample sizes. We believe that the integration of this framework in existing case-control studies, Biobanks and prospective cohorts would increase the yield of genes with pleiotropic effects and the identification of target populations for precision interventions.

## Figures and Tables

**Table 1:** List of disease/trait associated SNPs significantly differentiating between *Prakriti* groups as well as IE background control in NI and Vadu cohort. The risk allele frequency (RAF) of these SNPs in either of the *Prakriti* type is higher than the GWAS risk allele frequency, as shown in Figure S5A,B. Between the differentiating *Prakriti* groups, higher RAF is marked with **^##^**, lower with *.

Cohort	SNP	Gene	GWAS Disease/Trait	Risk Allele	Risk Allele Frequency (RAF)						Differentiating *Prakriti* Groups
					V	P	K	VPK Pooled	IE Control	GWAS	
Vadu	rs11603334 (5′UTR variation)	*ARAP1*	Fasting Blood Proinsulin levels	A	0.08 *	0.25	0.33 ^##^	0.22	0.12	0.25	V vs. C, V vs. K
	rs1552224	*ARAP1*	Acute insulin response	A	0.92 ^##^	0.72	0.67 *	0.77	0.88	NR	V vs. K, K vs. C
	rs3014246 (missense)	*CCDC17*	Apolipoprotein A1 levels	C	0.5 ^##^	0.39	0.19 *	0.36	0.50	0.29	V vs. K, K vs. C
	rs682331 (3′UTR variation)	*NIBAN1*	Obesity related traits	G	0.69 ^##^	0.27	0.2 *	0.39	0.41	0.44	V vs. C, V vs. P, V vs. K
	rs3811445 (synonymous)	*TRIM58*	Immature fraction of reticulocytes	G	0.58	0.79 ^##^	0.39 *	0.58	0.68	0.58	P vs. K, K vs. C
	rs10922162	*ASPM*	End-stage coagulation	C	0.72	0.56 *	0.81 ^##^	0.7	0.85	0.83	P vs. K, P vs. C
	rs1801222	*CUBN*	Homocysteine levels	A	0.31 ^##^	0.03 *	0.24 ^##^	0.19	0.09	0.34	P vs. K, V vs. P, V vs. C
	rs257377	*PRKAR2B*	LDL cholesterol	G	0.75 *	0.83	0.97 ^##^	0.85	0.71	0.79	V vs. K, K vs. C
	rs738409 (missense)	*PNPLA3*	Cirrhosis	G	0.28	0.08 *	0.36 ^##^	0.24	0.09	0.27	K vs. C, P vs. K
			Hb conc							0.21	
			Hb conc							0.26	
			Liver enzymes level							0.23	
			Liver fibrosis							0.21	
			Red cell distribution width							0.21	
			Total triglyceride levels							0.36	
			T2D							0.22	
NI	rs699 (nonsynonymous)	*AGT*	Mean Arterial Pressure	A	0.36 ^##^	0.25	0.11 *	0.24	0.38	0.48	V vs. K, K vs. C
	rs2792751 (nonsynonymous)	*GPAM*	HDL Cholesterol levels, Apolipoprotein A1 levels	T	0.16	0.37 ^##^	0.04 *	0.19	0.11	0.27	P vs. K, P vs. C
	rs3764002 (nonsynonymous)	*WSCD2*	T2D, Waist-to-hip ratio	C	0.83 ^##^	0.64	0.56 *	0.68	0.58	0.72,0.73	V vs. K, V vs. C
	rs3764002 (nonsynonymous)	*WSCD2*	Risk taking tendency, Predicted visceral adipose tissue	T	0.17 *	0.36	0.44 ^##^	0.32	0.41	0.26	V vs. K,V vs. C
	rs10793625 (5′UTR variant)	*WASH2C*	Mean corpuscular Hb levels	C	0.67 *	0.81	0.94 ^##^	0.81	0.61	0.79	V vs. K, K vs. C
	rs675531 (nonsynonymous)	*THEMIS*	Recalcitrant atopic dermatitis	C	0.43	0.66 ^##^	0.33 *	0.47	0.30	0.11	P vs. K, P vs. C
	rs8073060 (missense)	*SLFN14*	Platelet count	A	0.15 *	0.44 ^##^	0.35	0.31	0.44	0.29	V vs. P, V vs. C
	rs2073498 (missense)	*RASSF1*	Feeling worry	A	0.14	0.25 ^##^	0.06 *	0.15	0.05	0.11	P vs. K, P vs. C
	rs41269255 (nonsynonymous)	*POM121L2*	Depressive symptoms	T	0 *	0.08	0.21 ^##^	0.1	0.02	0.11	V vs. K, K vs. C
	rs17412833 (nonsynonymous)	*HLA-DQB1*	Lactate dehydrogenase levels	T	0.2 *	0.53 ^##^	0.38	0.37	0.52	0.13	V vs. P, V vs. C

## Data Availability

The datasets used and/or analyzed during the current study are available from the corresponding author on reasonable request.

## Supplementary Materials

The following supporting information can be downloaded at: https://www.mdpi.com/article/10.3390/jpm12030489/s1, Figure S1: Scatter plot of lowest *p* value from 10,000 permutations vs. 80,000 permutations in (A) NI (B) Vadu cohort, Figure S2: Distribution of significant SNPs across genomic regions in (A) NI (B) Vadu cohort, Figure S3: Barplot showing number of *Prakriti* differentiating SNPs associated with GWAS disease/traits Parent Term in pairwise *Prakriti* comparisons (VP, VK, PK) in (A) NI (B) Vadu cohort, Figure S4: Heatmap showing risk allele frequencies (RAF) for enriched disease/traits in GWAS catalog in (A) NI (B) Vadu cohort, Figure S5: Barplots for risk allele frequencies for GWAS SNPs mentioned in Table1, Figure S6: Barplot showing number of phenotypes from GeneATLAS associated (*p* < 0.05) with *Prakriti* differentiating genes in (A) NI (B) Vadu cohort, Figure S7: (a) Alternate allele “G” frequency bar plot of rs56084453 (*ZNF502*) across *Prakriti* groups, IE Control and Pooled population (V, P, K combined) in both cohorts. Frequency of the alternate allele “G” is significantly lower in the *Pitta* group than in *Vata* and *Kapha* (*p* < 0.04416, *p* < 0.01932 NI cohort; *p* < 0.02493, *p* < 0.002212 Vadu cohort). This SNP also significantly differs from background control in Vadu cohort (*p* < 0.004488). Violin plots of rs56084453 *(ZNF502)* acting as eQTL in (b) Whole Blood (c) Spleen from GTEx v8, Figure S8: Box plot showing power for significant SNPs with variable sample sizes (original sample size, 18, 50, 100, 500, 1000, 10,000) in *Prakriti* comparison in (A) NI (B) Vadu cohort, Table S1: *Prakriti* differentiating SNPs post permutation testing with *p* < 0.05 in NI & Vadu cohort, Table S2: List of enriched Biological Processes (*p* < 0.01) from Toppfunn in VP, VK, PK in NI and Vadu cohorts, Table S3: *Prakriti* differentiating SNPs associated with diseases/traits in GWAS catalog, Table S4: *Prakriti* differentiating SNPs in NI and Vadu cohort acting as cis-eQTL in various tissues in GTEx v8, Table S5: Results for enrichment analysis (*p* < 0.05) for GWAS diseases/traits in V, P, K groups in NI and Vadu cohort, Table S6: *Prakriti* differentiating SNPs associated with UK Biobank phenotypes (*p* < 0.05) in GeneATLAS in NI and Vadu cohort, Table S7: Replication of *Prakriti* differentiating Genes & SNPs between NI and Vadu cohort, Table S8: Replication of *Prakriti* differentiating SNPs with similar profiles between NI and Vadu cohort, Table S9: List of replicated profile SNPs acting as cis-eQTL in various GTEx tissues, Table S10: Power Analysis results with increasing sample size for *Prakriti* differentiating SNPs in NI and Vadu cohort.
