# Supplementary material for: Whole Exome Sequencing in Healthy Individuals of Extreme Constitution Types Reveals Differential Disease Risk: A Novel Approach towards Predictive Medicine
Source: J Pers Med. 2022 Mar 18;12(3):489. doi: 10.3390/jpm12030489 (PMC8952204; doi:10.3390/jpm12030489)
Supplement: Supplementary file 1 [file jpm-12-00489-s001.zip › jpm-1618876-supplementary/Supplementary Material/Supplementary Figures-20211119T162433Z-001/Supplementary Figures/Fig S1.pdf]

(A)

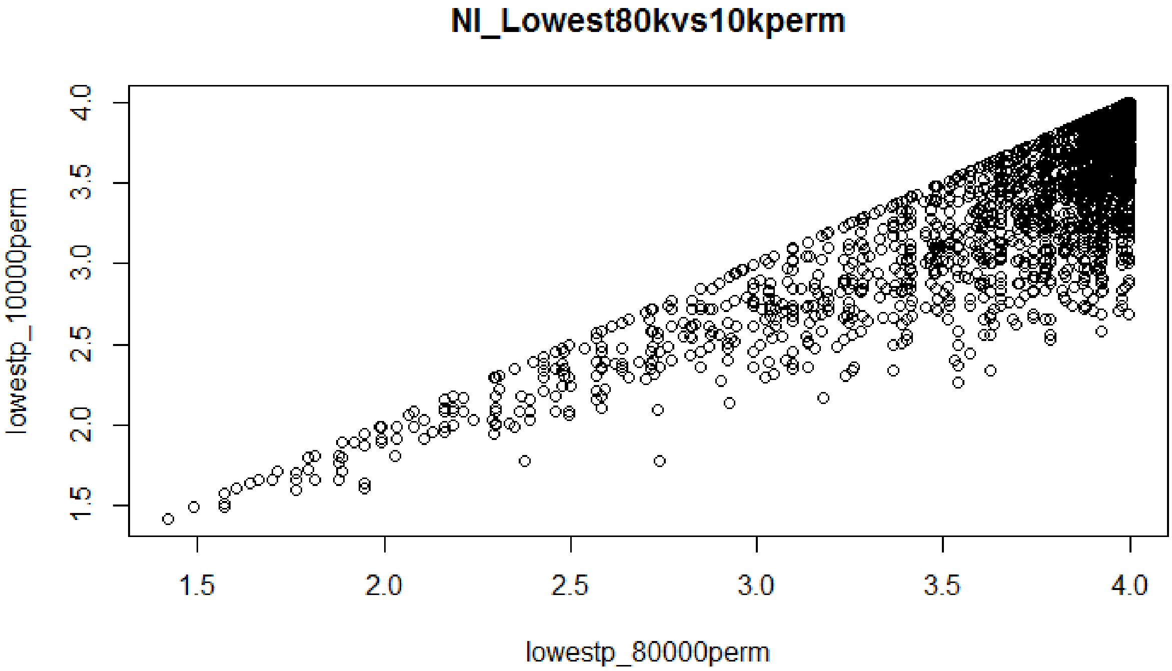

NI_Lowest80kvs10kperm
lowestp_10000perm
1.5
2.0
2.5
3.0
3.5
4.0
lowestp_80000perm

(B)

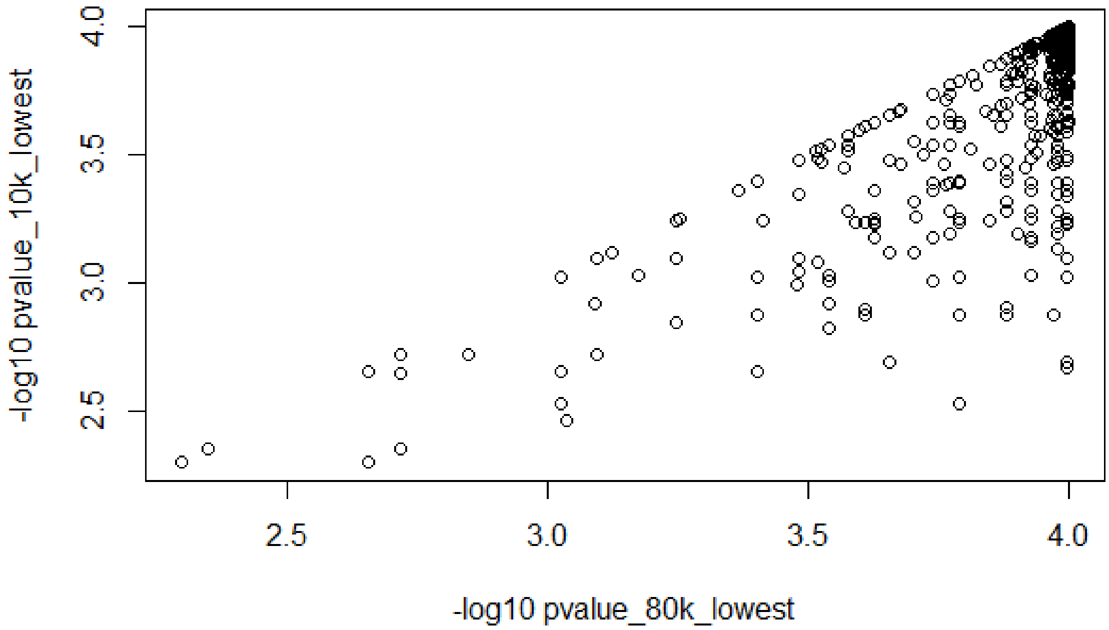

-log10 pvalue_10k_lowest
2.5
3.0
3.5
4.0
-log10 pvalue_80k_lowest

Supp Figure S1 : Scatter plot for lowest p value from 80000 permutation set vs 10000 permutation set in (A) NI (B) Vadu cohort
