# Supplementary figures and images for: Whole Exome Sequencing in Healthy Individuals of Extreme Constitution Types Reveals Differential Disease Risk: A Novel Approach towards Predictive Medicine
Source: J Pers Med. 2022 Mar 18;12(3):489. doi: 10.3390/jpm12030489 (PMC8952204; doi:10.3390/jpm12030489)

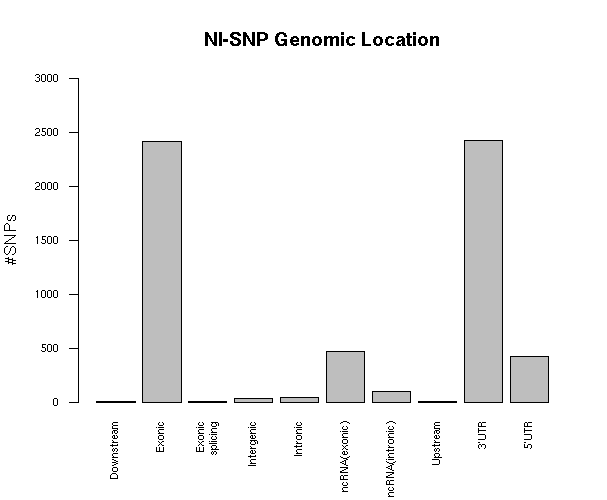

Supplement: Supplementary file 1 [file jpm-12-00489-s001.zip › jpm-1618876-supplementary/Supplementary Material/Supplementary Figures-20211119T162433Z-001/Supplementary Figures/Fig S2(A) NI-SNP-Location.png]

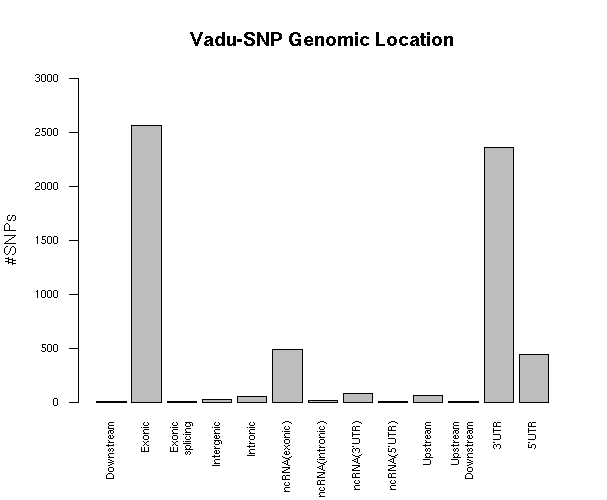

Supplement: Supplementary file 1 [file jpm-12-00489-s001.zip › jpm-1618876-supplementary/Supplementary Material/Supplementary Figures-20211119T162433Z-001/Supplementary Figures/Fig S2(B) Vadu-SNP-Location.png]

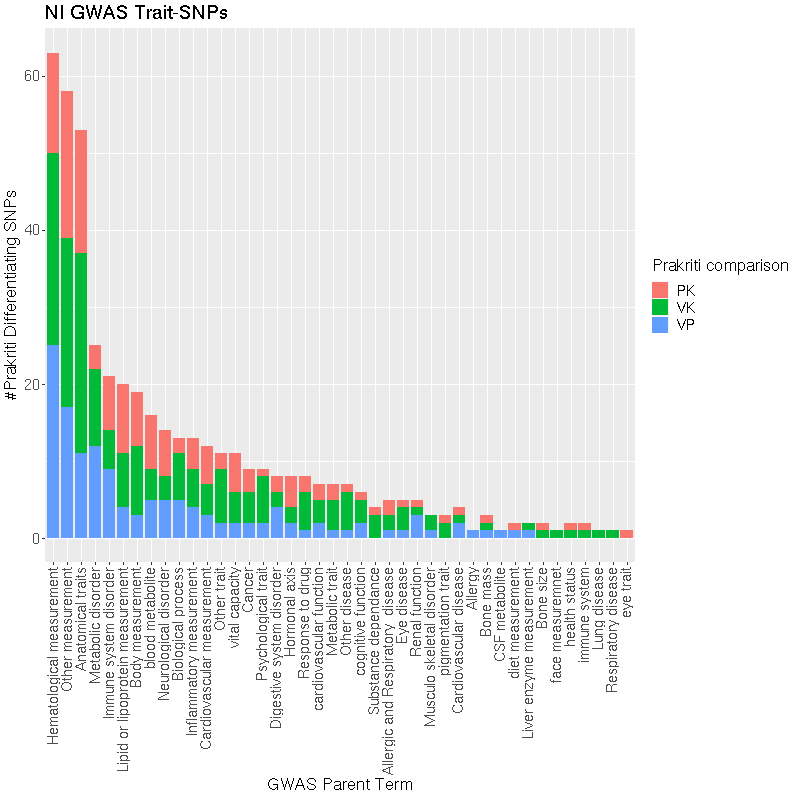

Supplement: Supplementary file 1 [file jpm-12-00489-s001.zip › jpm-1618876-supplementary/Supplementary Material/Supplementary Figures-20211119T162433Z-001/Supplementary Figures/Fig S3(A) NI_GWAS_Trait_frq.png]

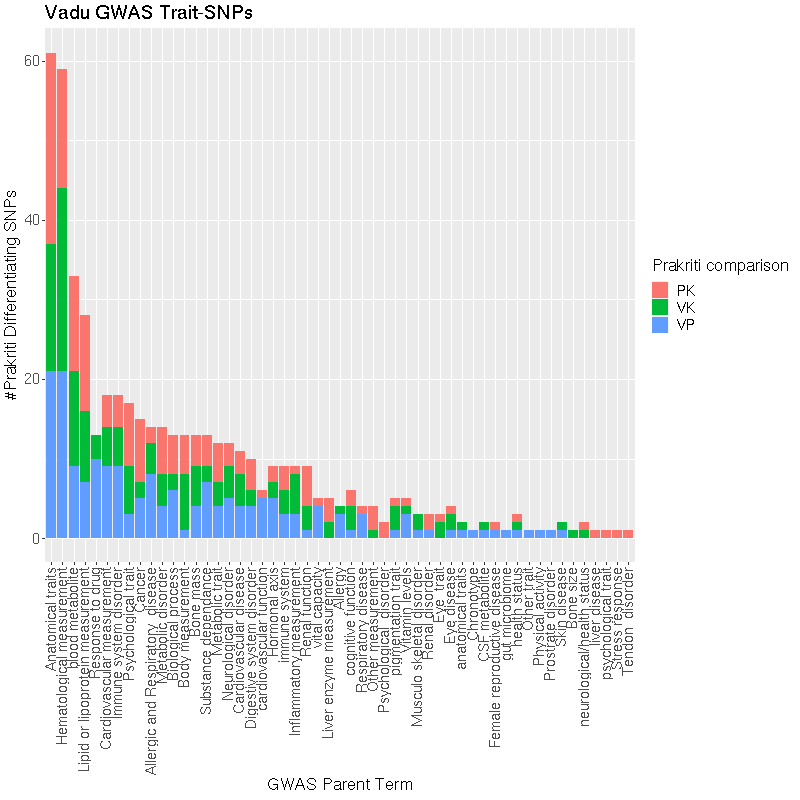

Supplement: Supplementary file 1 [file jpm-12-00489-s001.zip › jpm-1618876-supplementary/Supplementary Material/Supplementary Figures-20211119T162433Z-001/Supplementary Figures/Fig S3(B) Vadu_GWAS_Trait_frq.png]

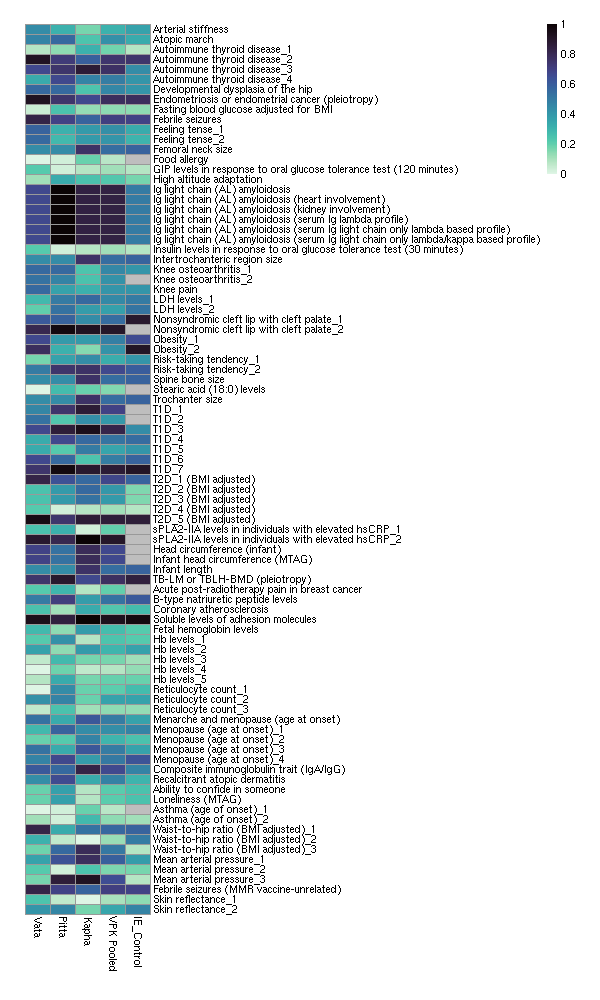

Supplement: Supplementary file 1 [file jpm-12-00489-s001.zip › jpm-1618876-supplementary/Supplementary Material/Supplementary Figures-20211119T162433Z-001/Supplementary Figures/Fig S4(A) NI_heatmap_enricheddata.png]

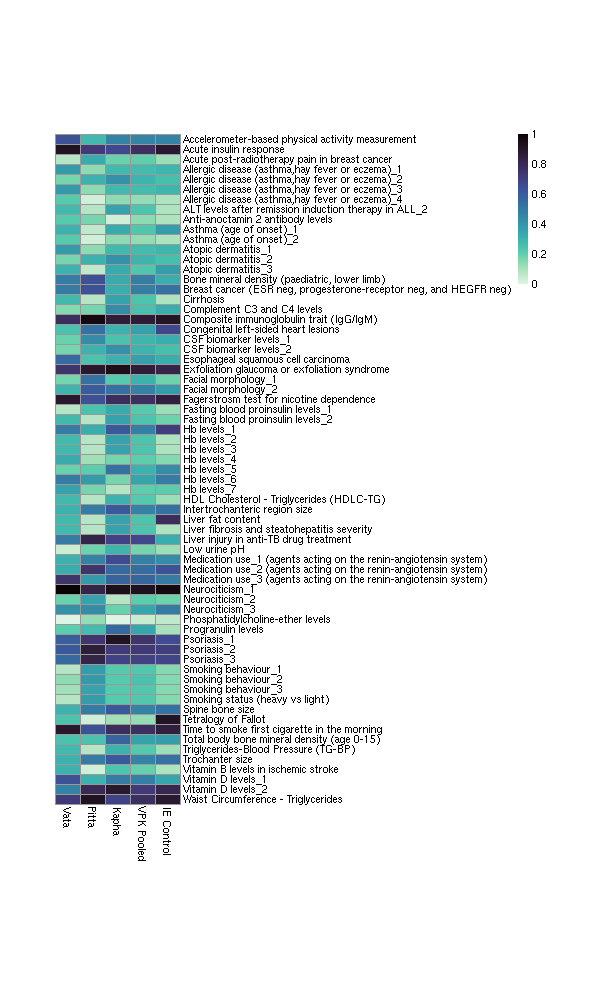

Supplement: Supplementary file 1 [file jpm-12-00489-s001.zip › jpm-1618876-supplementary/Supplementary Material/Supplementary Figures-20211119T162433Z-001/Supplementary Figures/Fig S4(B) Vadu_heatmap_enricheddata.png]

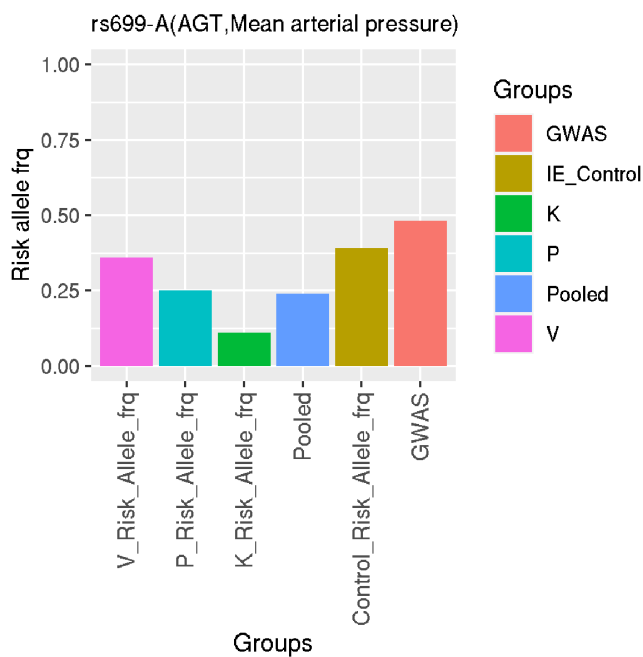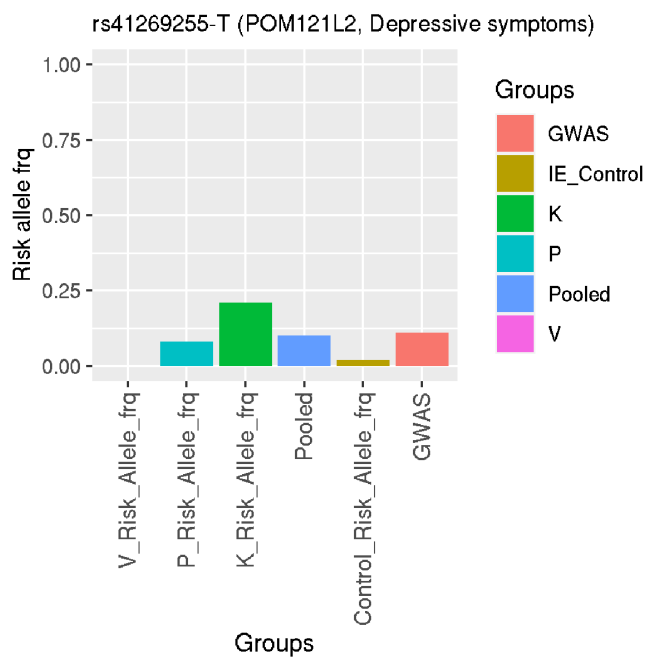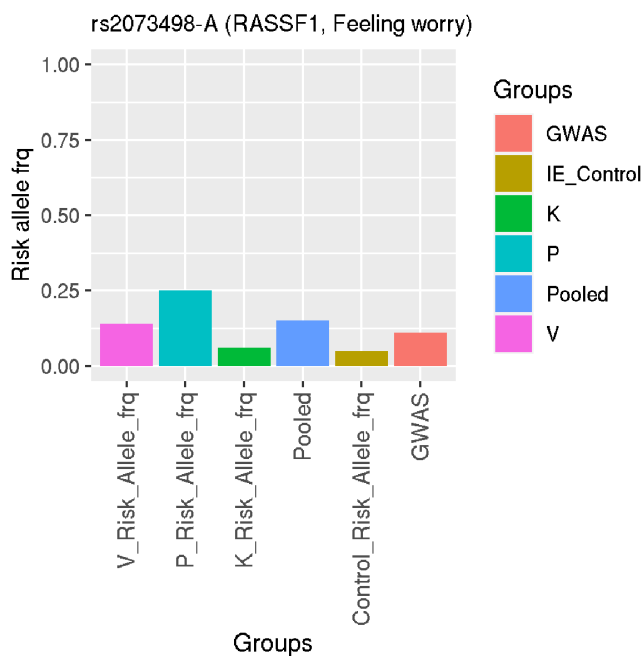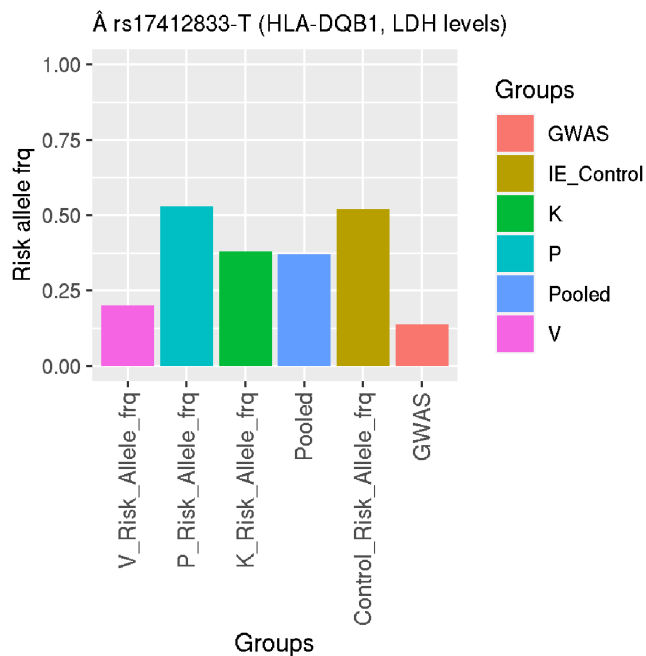

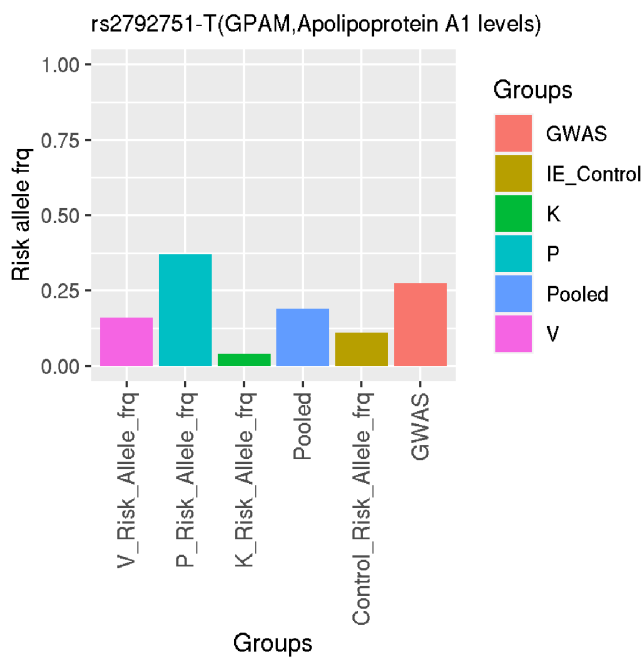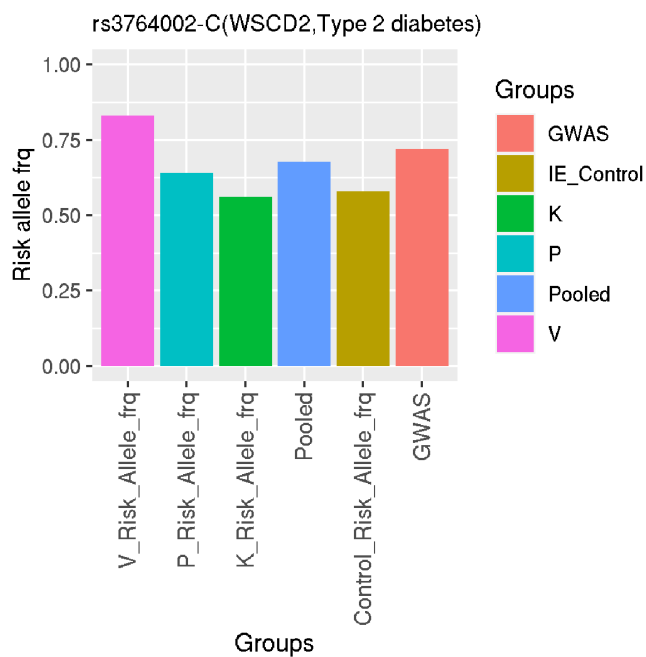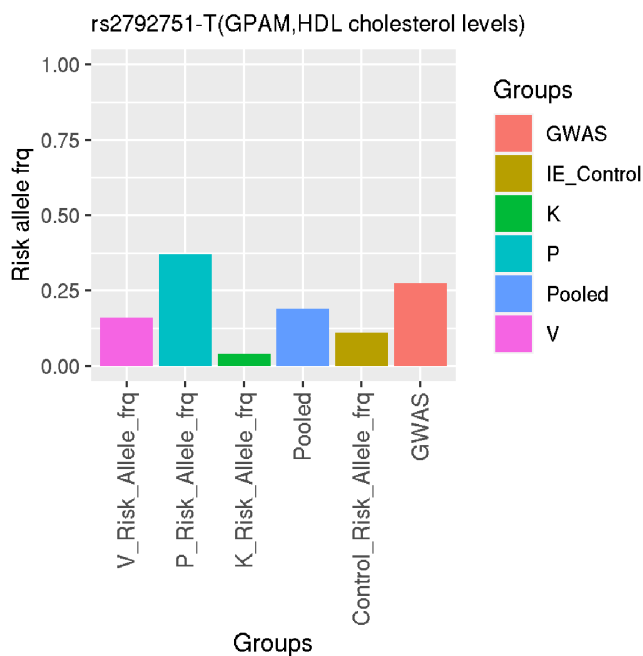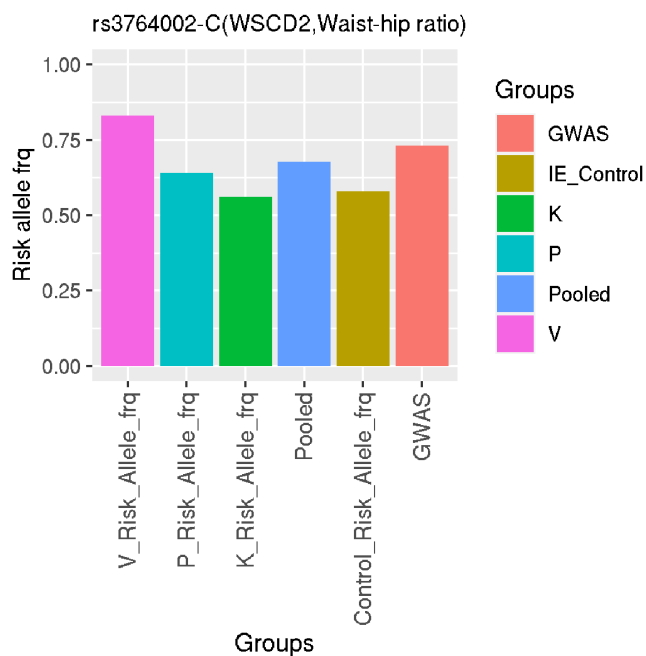

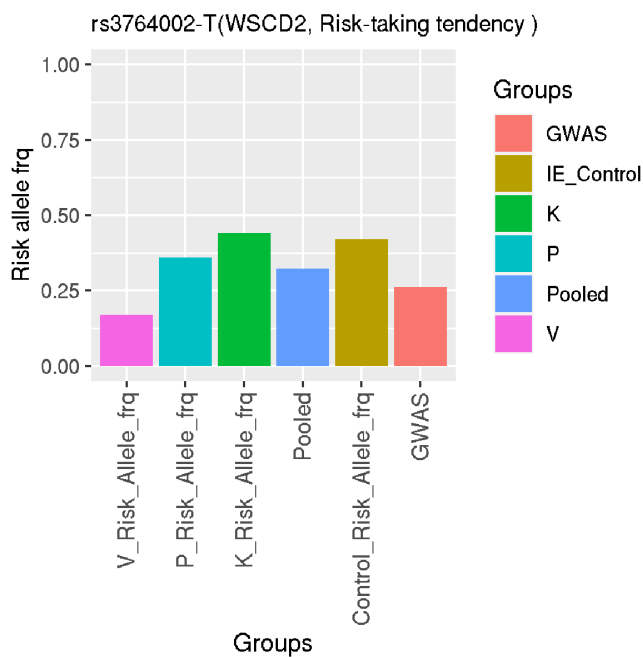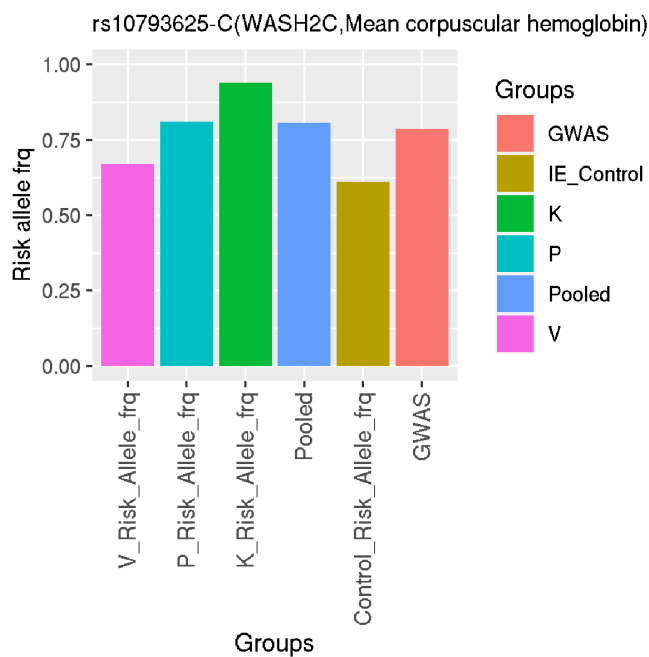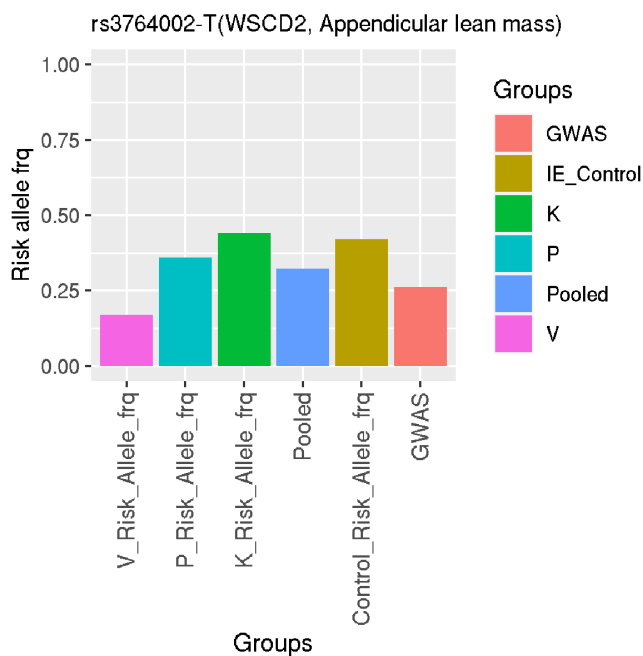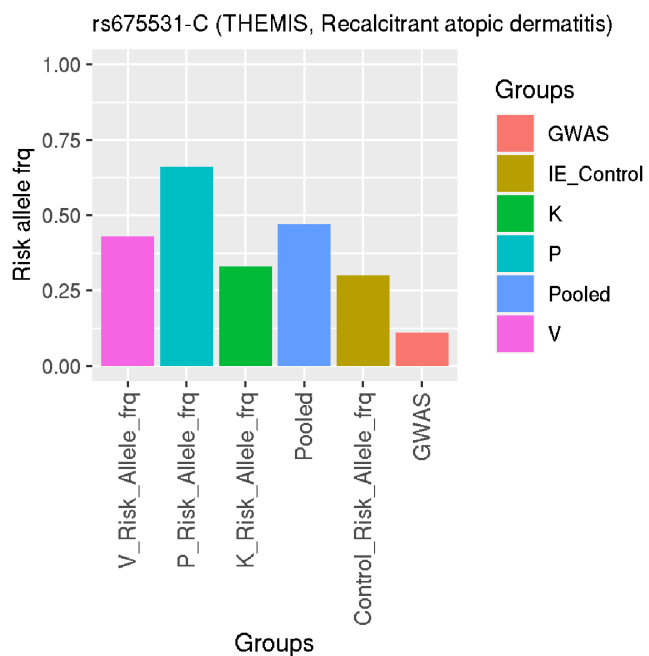

Supplement: Supplementary file 1 [file jpm-12-00489-s001.zip › jpm-1618876-supplementary/Supplementary Material/Supplementary Figures-20211119T162433Z-001/Supplementary Figures/Fig S5(A) NI-Heatmap_barplots.pdf]

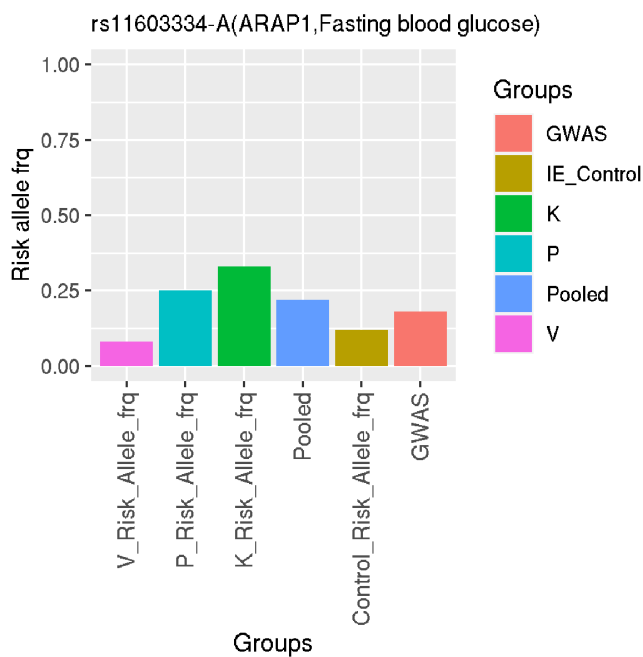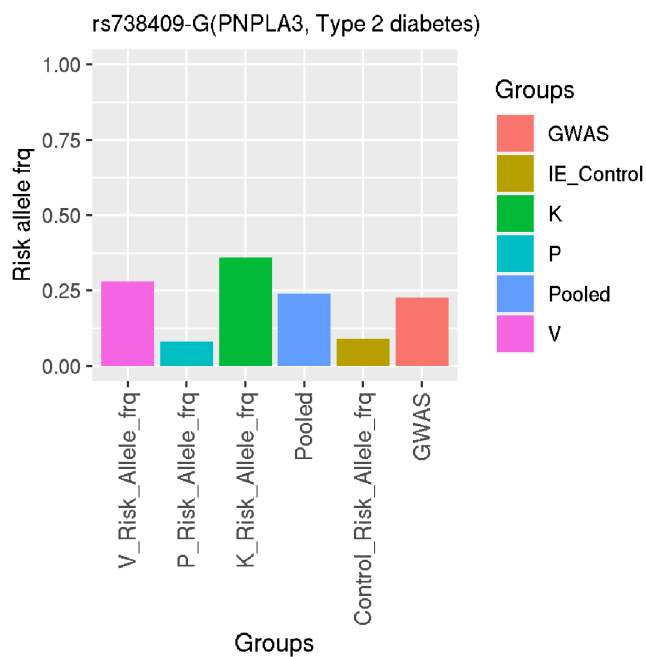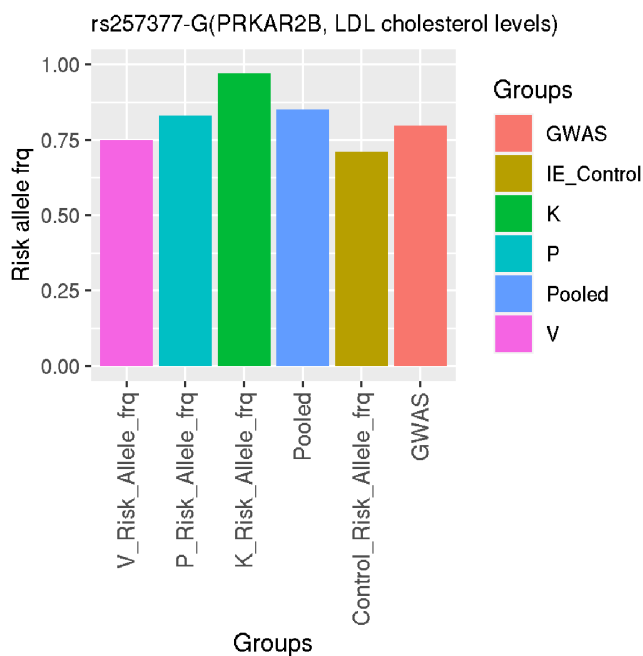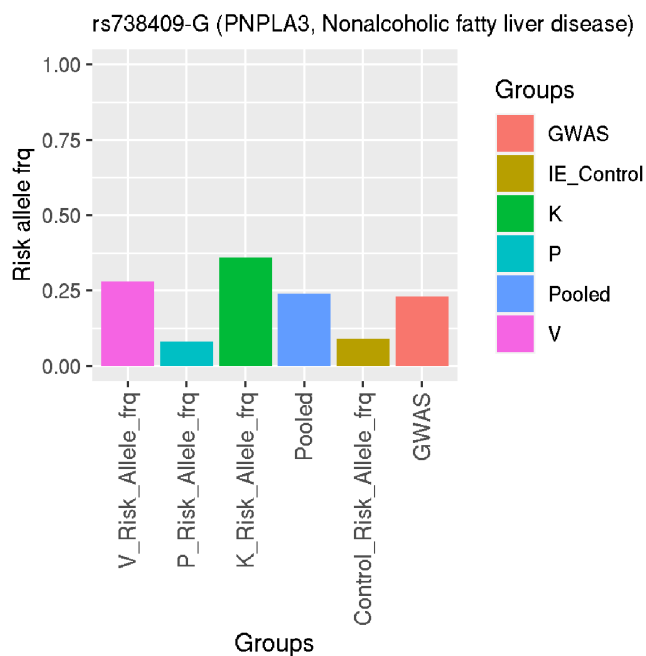

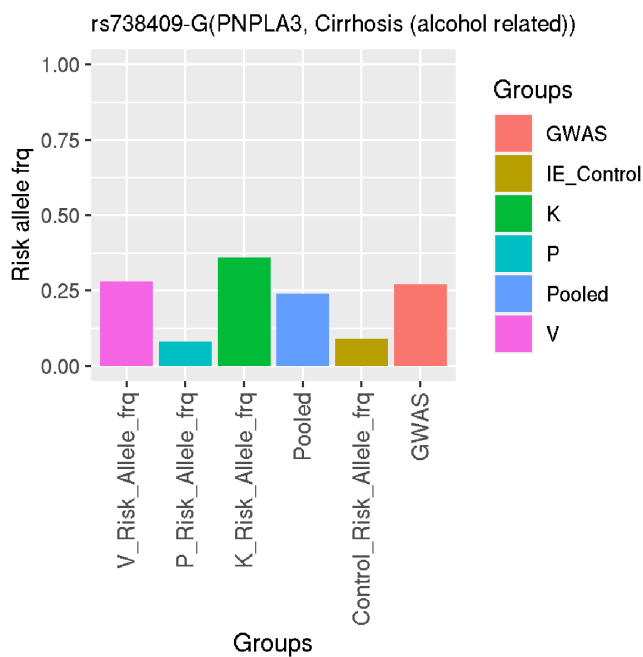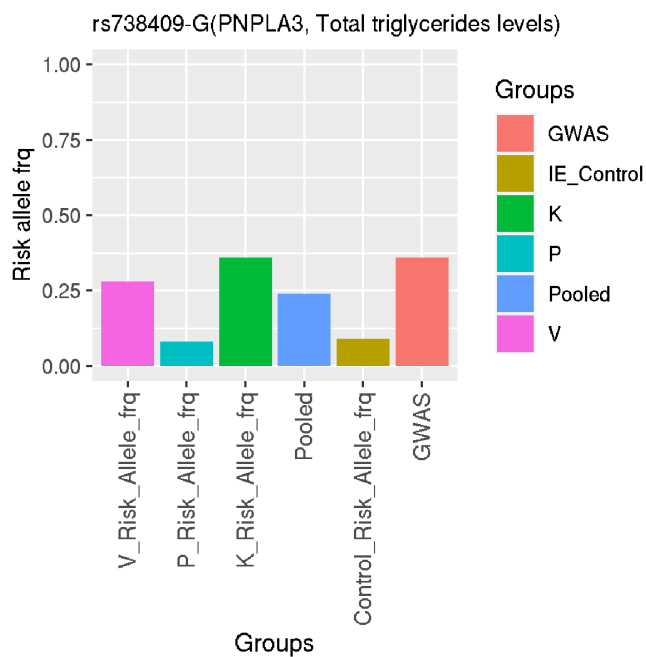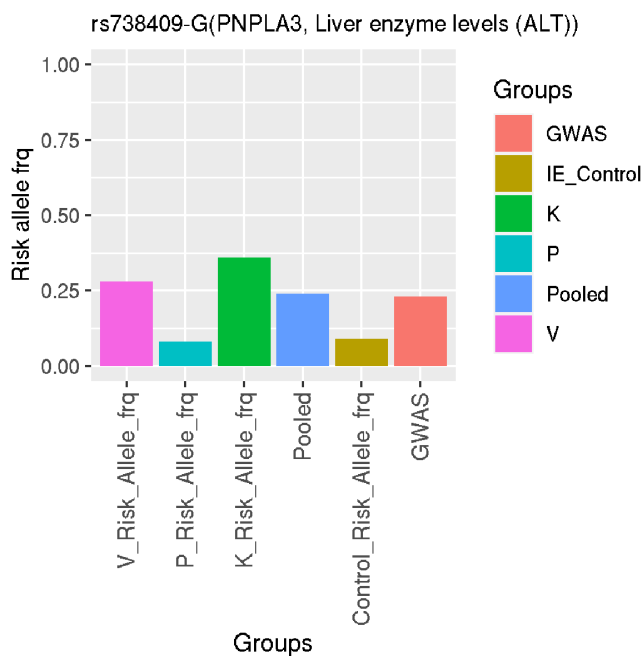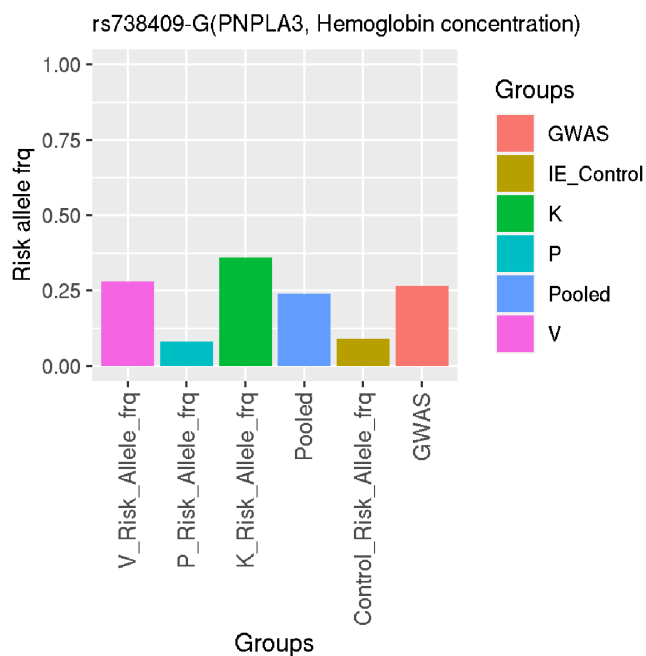

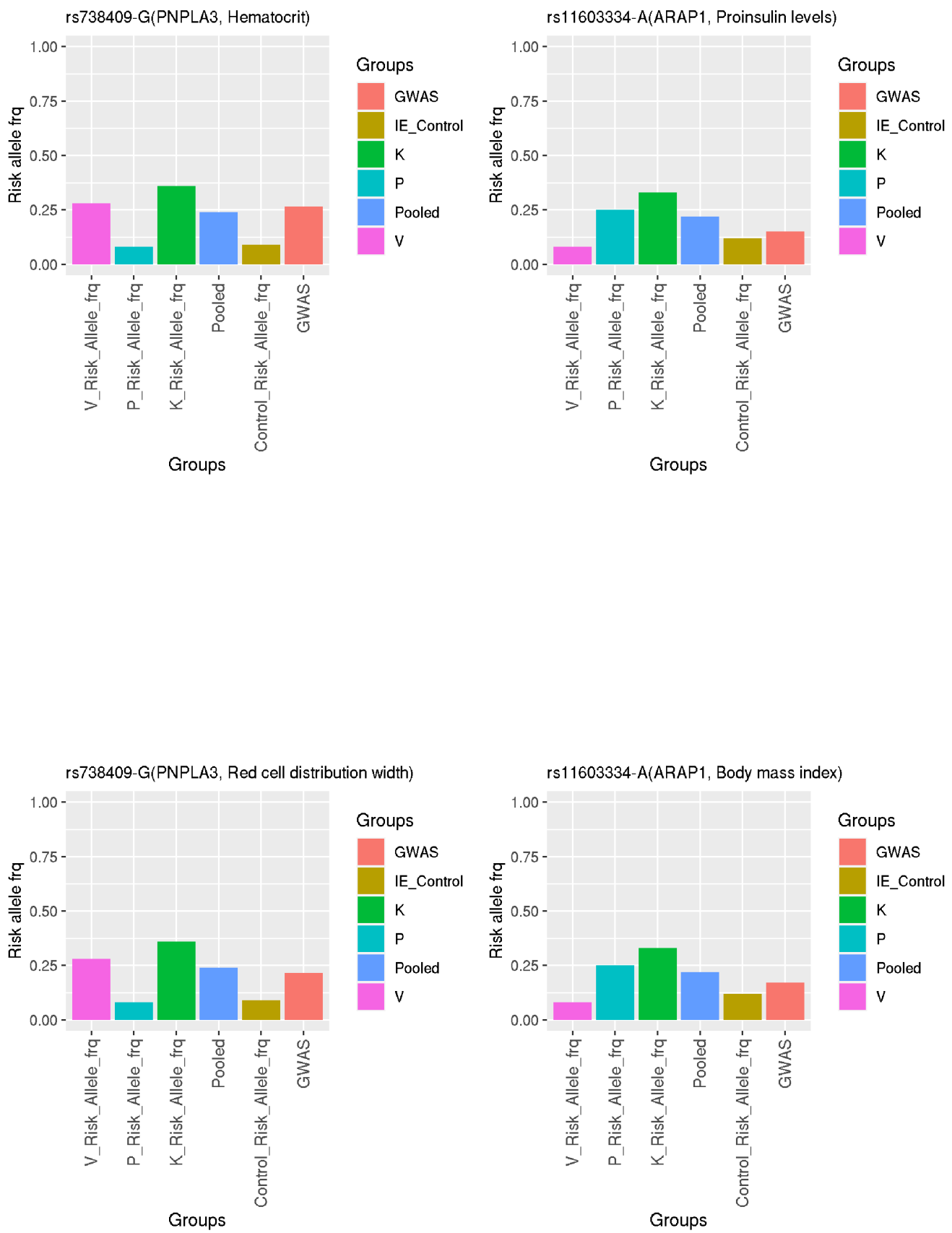

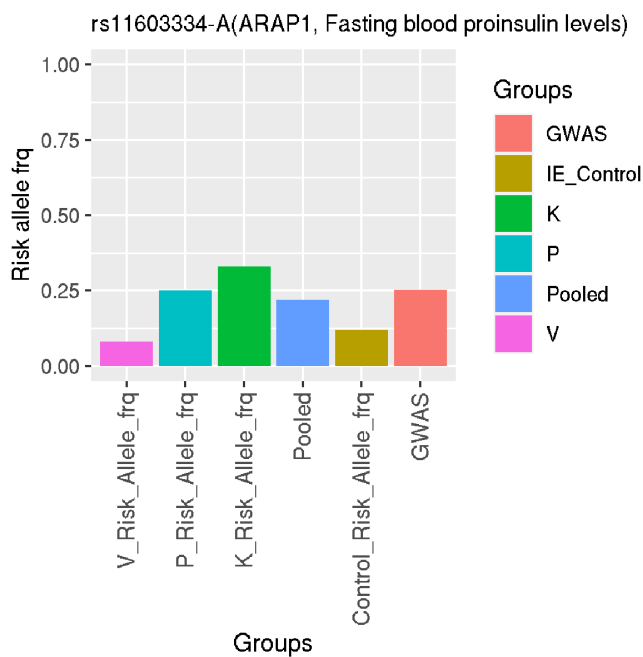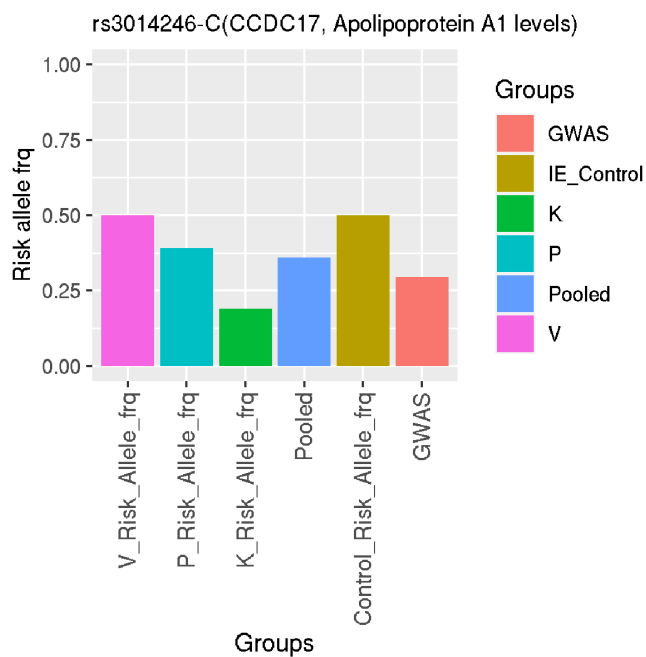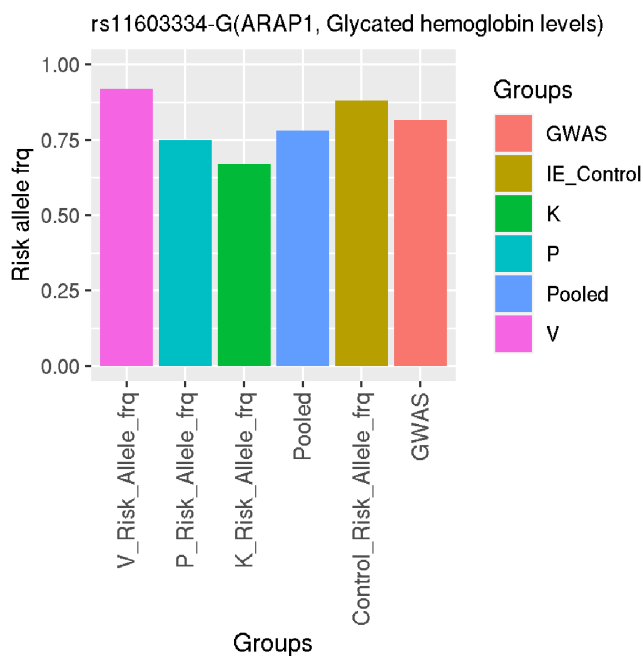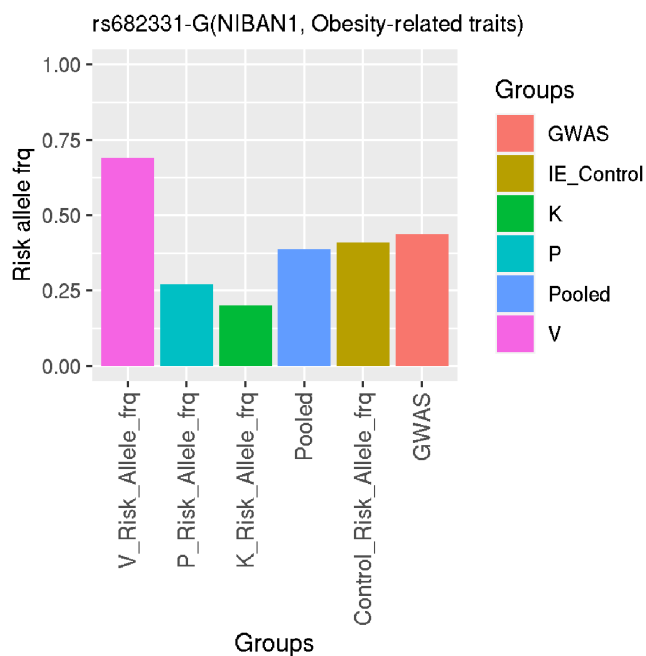

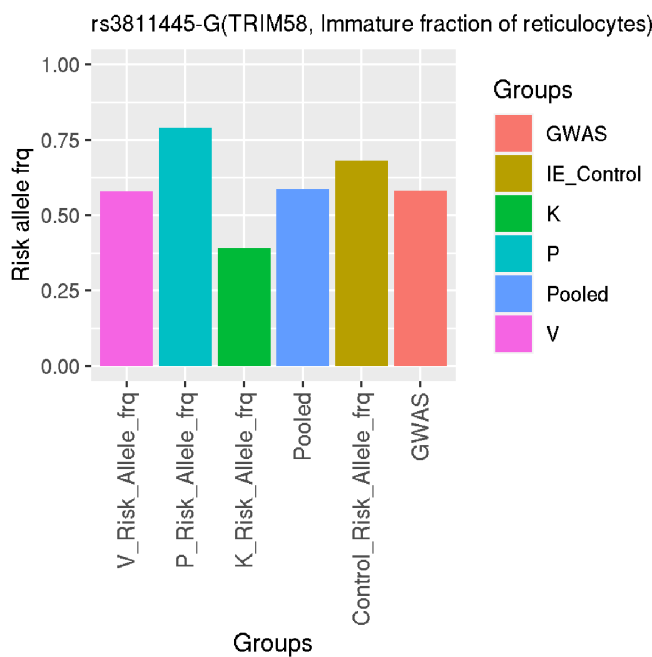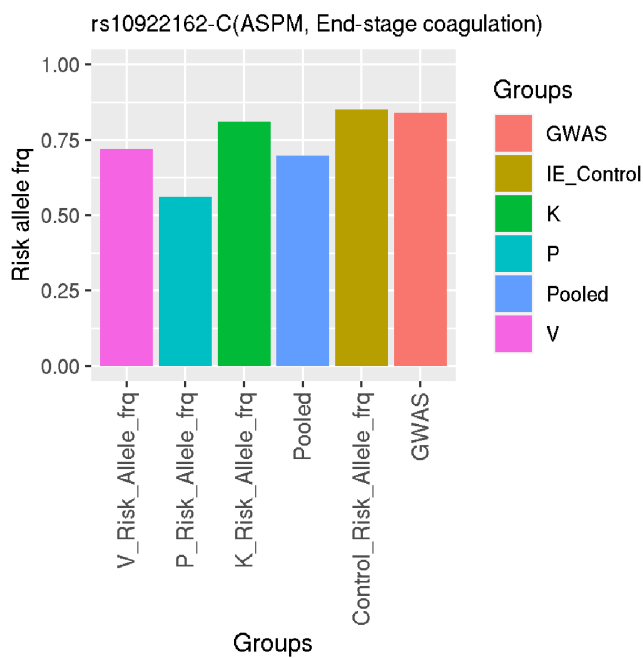

Supplement: Supplementary file 1 [file jpm-12-00489-s001.zip › jpm-1618876-supplementary/Supplementary Material/Supplementary Figures-20211119T162433Z-001/Supplementary Figures/Fig S5(B) Vadu-Heatmap_barplots.pdf]

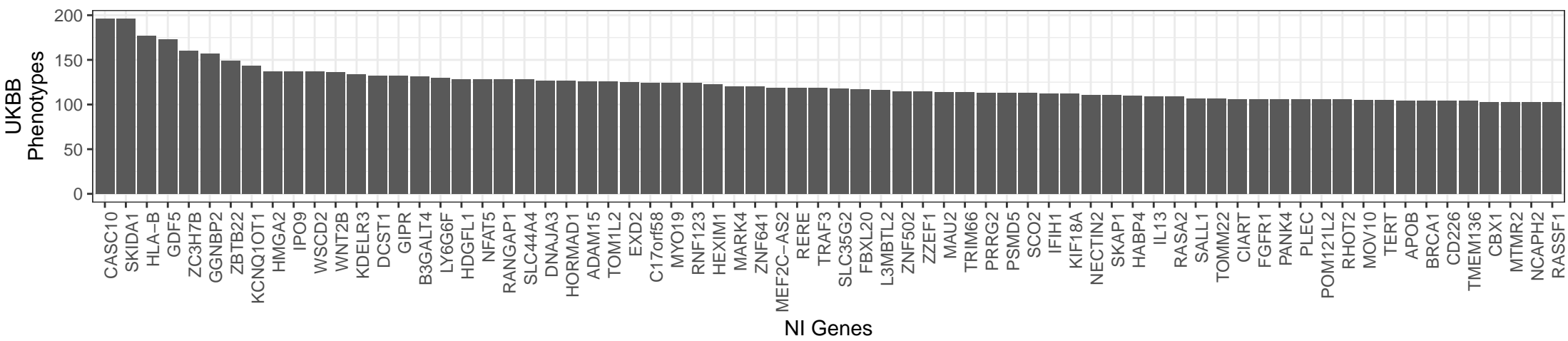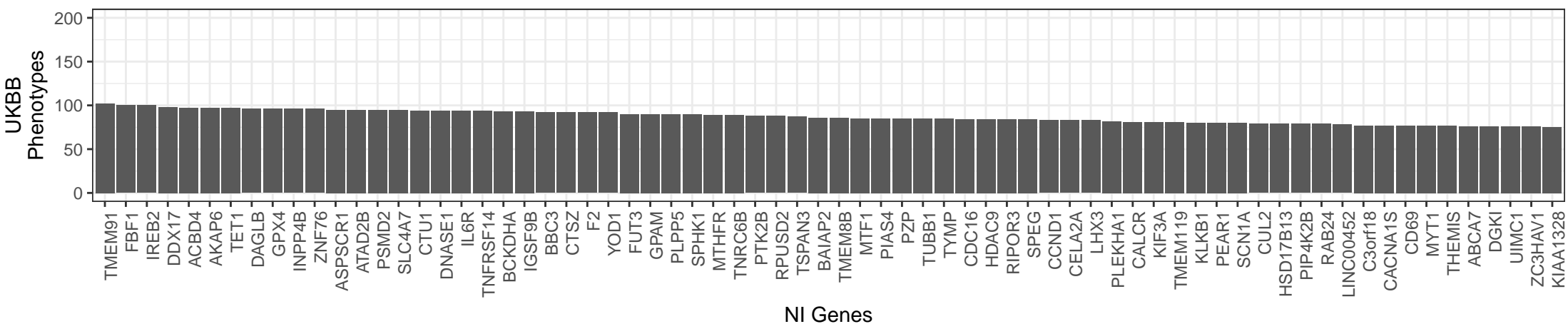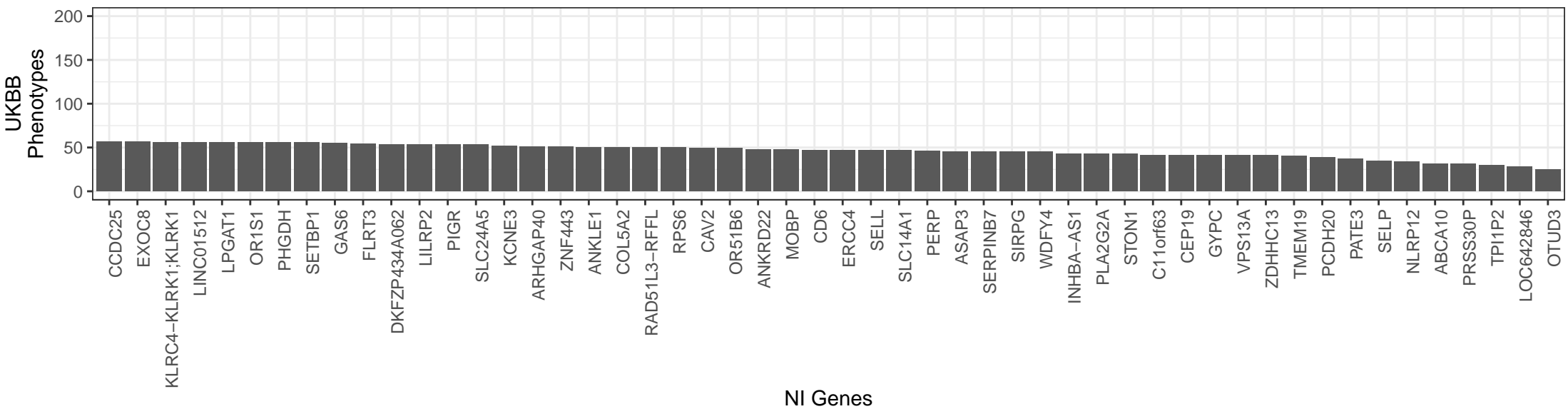

Supplement: Supplementary file 1 [file jpm-12-00489-s001.zip › jpm-1618876-supplementary/Supplementary Material/Supplementary Figures-20211119T162433Z-001/Supplementary Figures/Fig S6(A) NI_UKBB_Gene_Pheno.pdf]

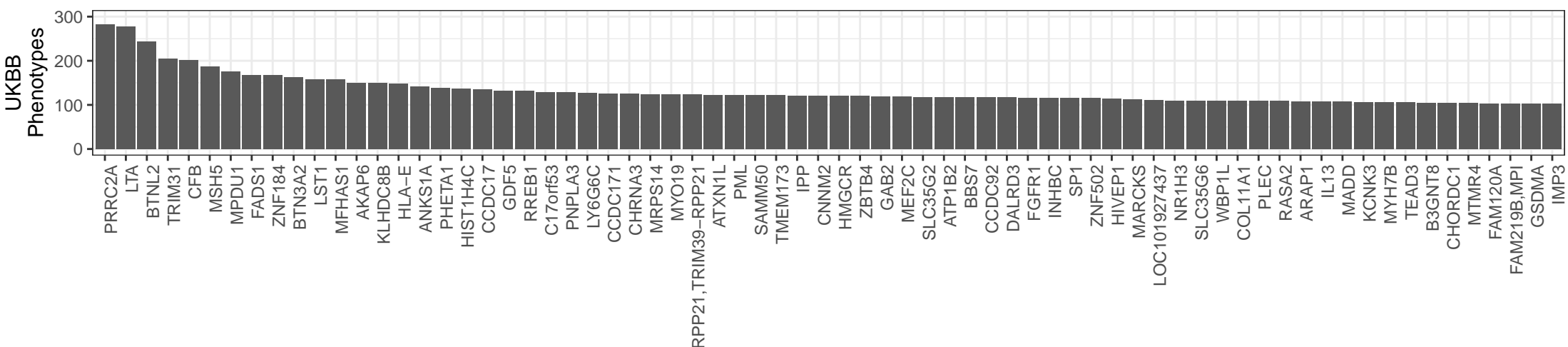

Vadu Genes

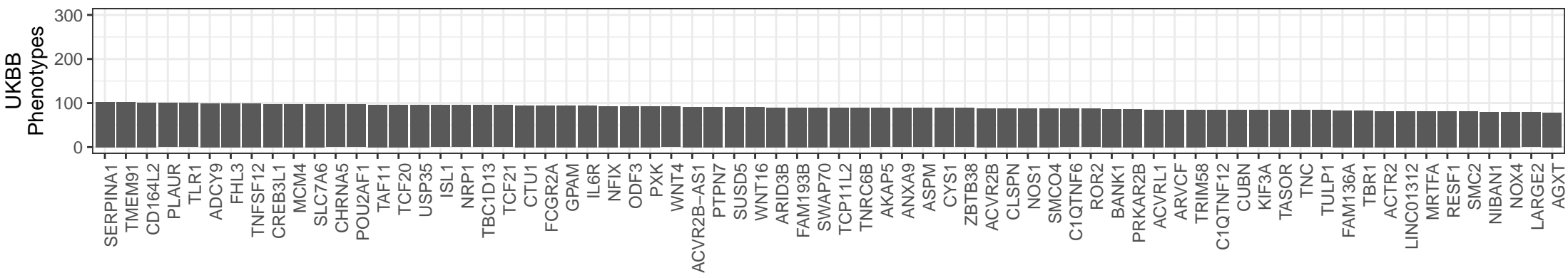

Vadu Genes

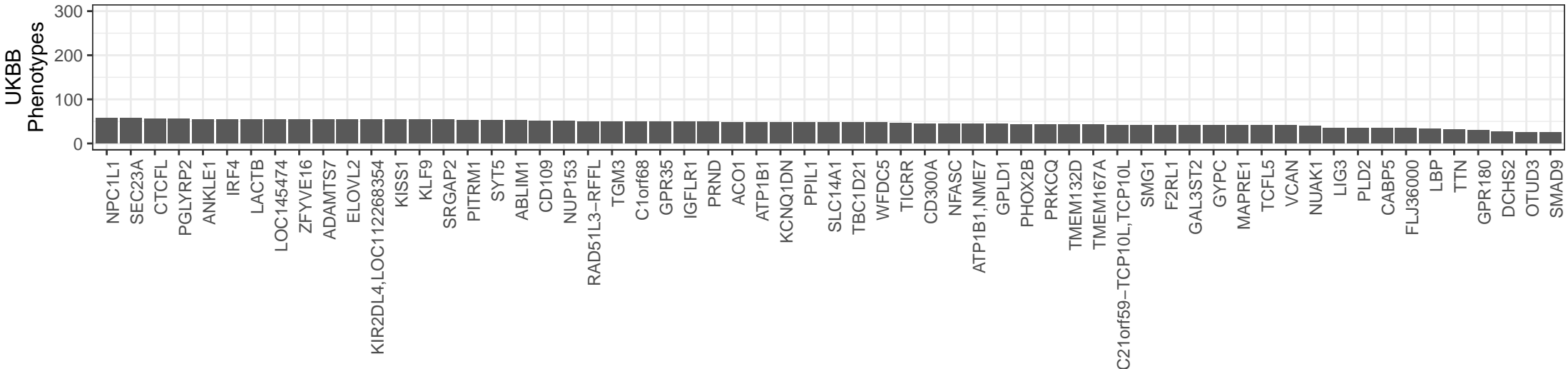

Vadu Genes

Supplement: Supplementary file 1 [file jpm-12-00489-s001.zip › jpm-1618876-supplementary/Supplementary Material/Supplementary Figures-20211119T162433Z-001/Supplementary Figures/Fig S6(B) Vadu_UKBB_Gene_Pheno.pdf]

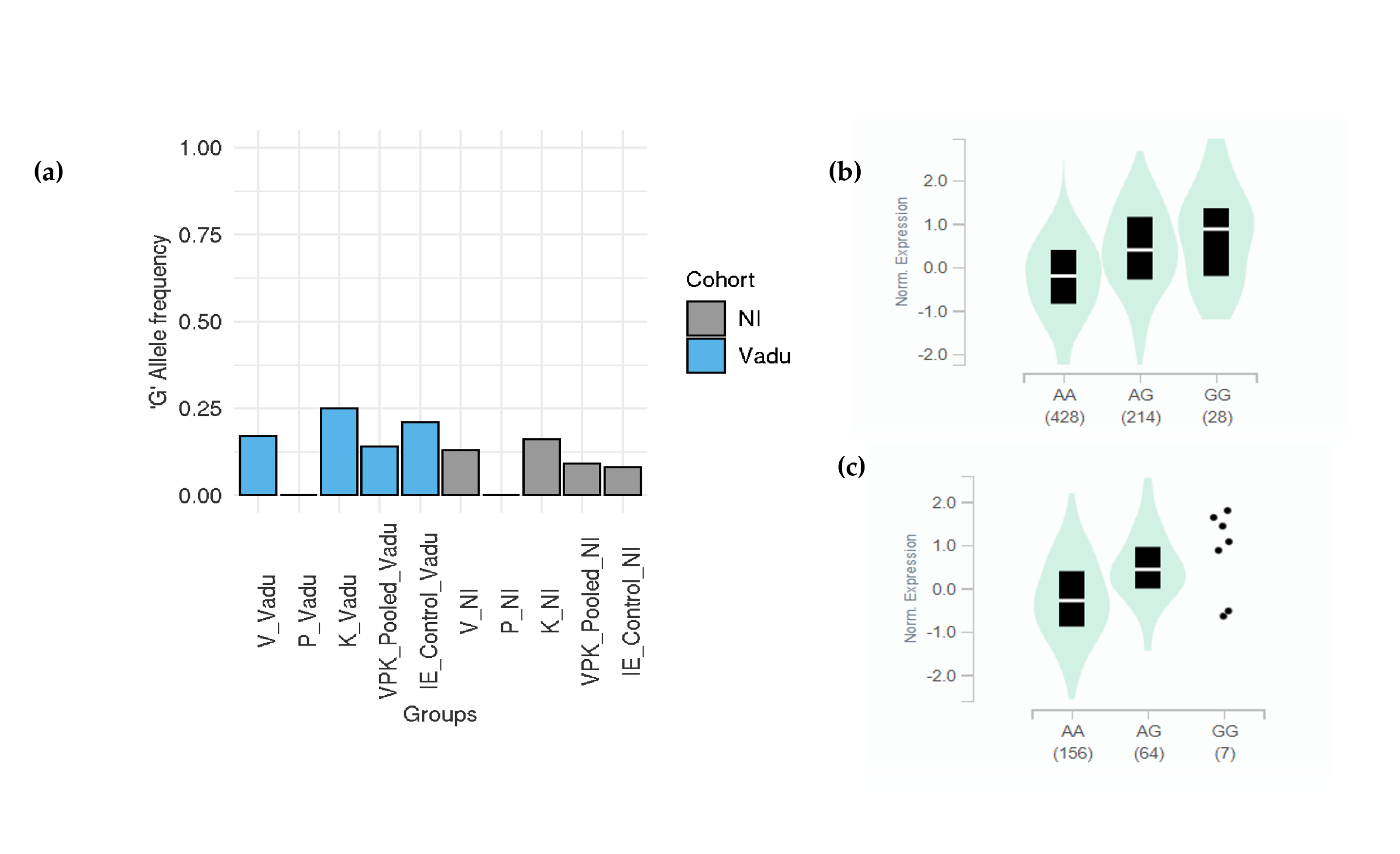

Supplement: Supplementary file 1 [file jpm-12-00489-s001.zip › jpm-1618876-supplementary/Supplementary Material/Supplementary Figures-20211119T162433Z-001/Supplementary Figures/Fig S7-ZNF502 Allelefrq Barplot & GTEx plots.png]

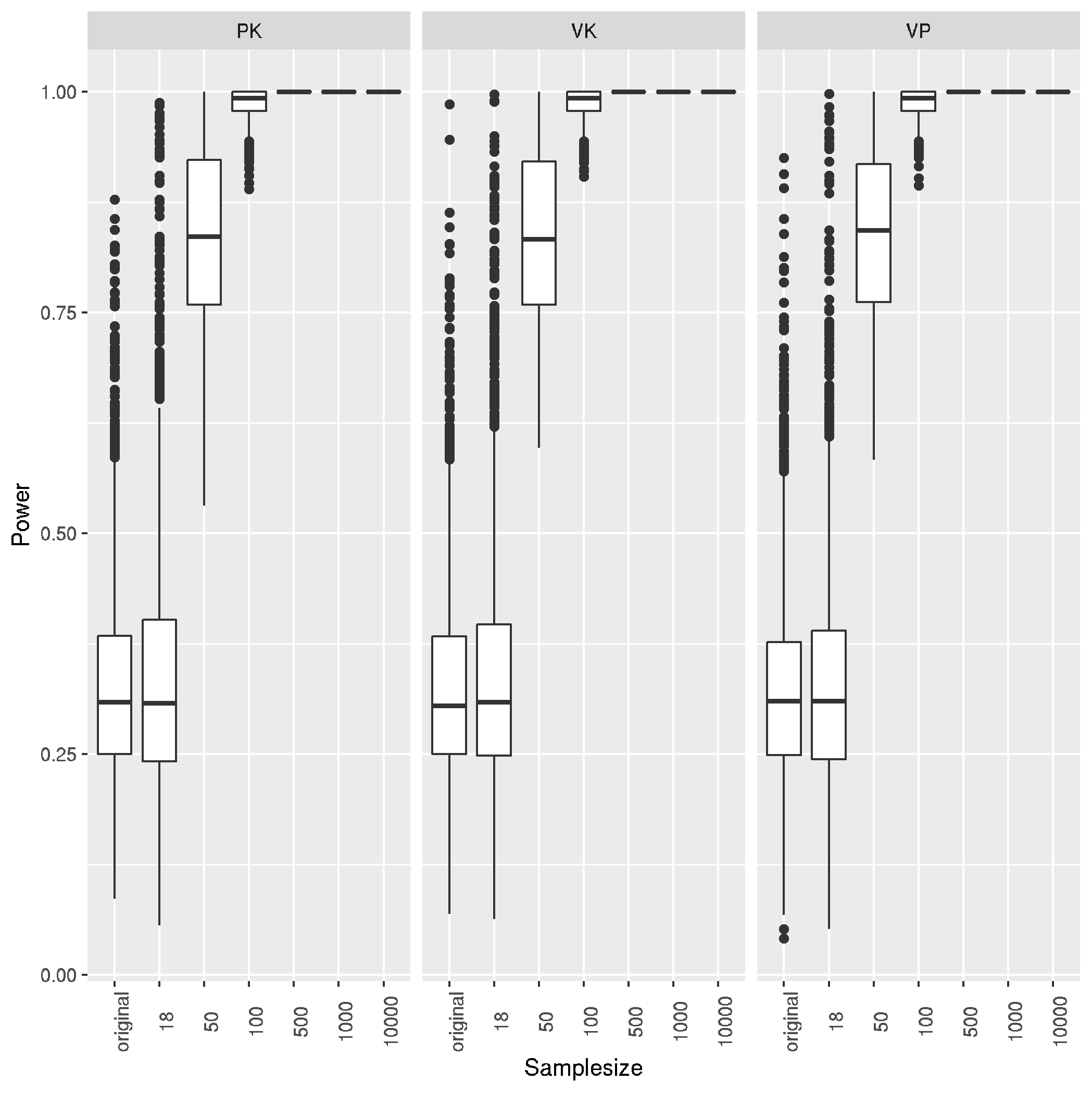

Supplement: Supplementary file 1 [file jpm-12-00489-s001.zip › jpm-1618876-supplementary/Supplementary Material/Supplementary Figures-20211119T162433Z-001/Supplementary Figures/Fig S8(A) NI_Power.png]

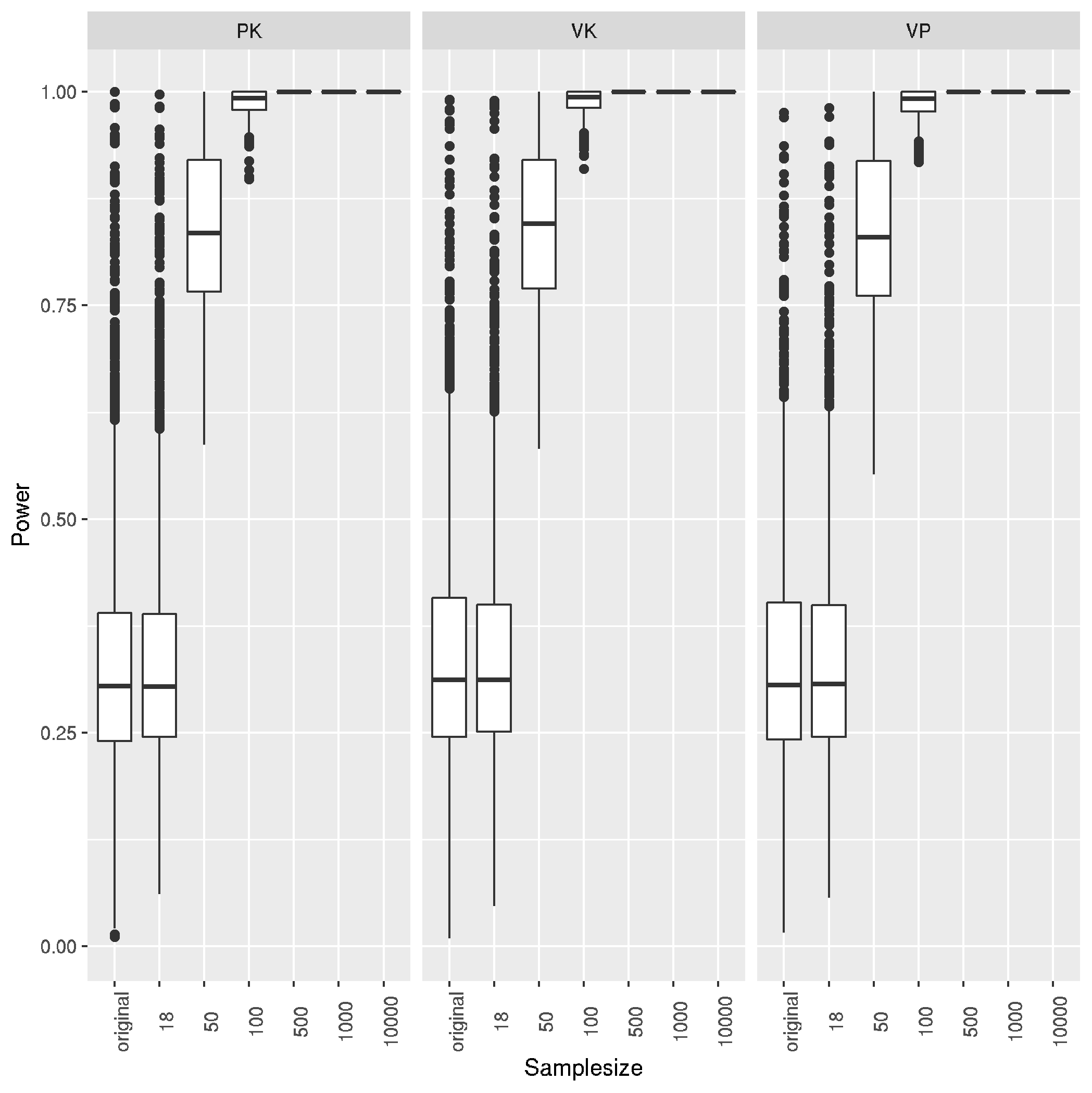

Supplement: Supplementary file 1 [file jpm-12-00489-s001.zip › jpm-1618876-supplementary/Supplementary Material/Supplementary Figures-20211119T162433Z-001/Supplementary Figures/Fig S8(B) Vadu_Power.png]
